# Late-Stage Presentation and Diagnostic Gaps in Romanian Lung Cancer Patients: A Retrospective Tertiary Center Analysis with Implications for Scalable Screening

**DOI:** 10.3390/diagnostics16172849

**Published:** 2026-09-04

**Authors:** Liviu Bîlteanu, Antonia-Ruxandra Folea, Vlad-Luca Moga, Bogdan Cosmin Tănase, Steluța Bărăscu, Cristian Pavel, Diana Troncotă, Matei Celea, Octavian Buiu, Andreea-Iren Șerban, Rodica Anghel

**Affiliations:** 1 Faculty of Biology, University of Bucharest, 91-95 Splaiul Independentei, 050095 Bucharest, Romania; liviu.bilteanu@umfcd.ro (L.B.); andreea-iren.serban@fmvb.usamv.ro (A.-I.Ș.); 2 Faculty of General Medicine, Carol Davila University of Medicine and Pharmacy, 8 Eroii Sanitari Street, 050474 Bucharest, Romania; vlad-luca.moga@drd.umfcd.ro (V.-L.M.); cristian-pavel.pavel@drd.umfcd.ro (C.P.); diana.troncota@drd.umfcd.ro (D.T.); mateicelea@gmail.com (M.C.); rodica.anghel@umfcd.ro (R.A.); 3 Laboratory for Molecular Nanotechnologies, National Institute for Research and Development in Microtechnologies—IMT Bucharest, 126A, Erou Iancu Nicolae Street, 077190 Voluntari, Romania; octavian.buiu@imt.ro; 4 Oncological Institute “Alexandru Trestioreanu” Bucharest, 252 Soseaua Fundeni, 022328 Bucharest, Romania; bogdansen@gmail.com (B.C.T.); stelutahuzuu@yahoo.com (S.B.); 5 Department of Preclinical Sciences, Faculty of Veterinary Medicine, University of Agronomic Sciences and Veterinary Medicine, 105 Splaiul Independentei, 050097 Bucharest, Romania

**Keywords:** lung cancer, lung cancer screening, early detection, late-stage presentation, diagnostic gap, epidemiology

## Abstract

**Background/Objectives:** Lung cancer in Romania is characterized by high mortality and a lack of organized screening programs, resulting in frequent late-stage diagnoses. This study evaluates diagnostic patterns, stage distribution, and screening eligibility in a Romanian tertiary cohort to highlight the limitations of standard screening criteria and provide evidence for scalable, regionally adapted early detection strategies. **Methods:** We conducted a retrospective observational study of consecutive lung cancer patients treated at the Oncological Institute of Bucharest. To balance external validity with analytical robustness, the population was divided into a full cohort (Set A, n = 3764) for population-level descriptive analyses and a complete-case subset (Set B, n = 1768). Set B was utilized to simulate theoretical screening eligibility using established guidelines (USPSTF, NCCN, ERS) and to calculate a Missed Opportunity Index (MOI) for early-stage case capture. **Results:** Advanced-stage disease (Stages III–IV) overwhelmingly dominated the cohort, accounting for approximately 82% of diagnoses among patients with available staging information. Essential screening variables like smoking history were missing in 71.4% of the full cohort. In the complete-case subset, established guideline-based screening models demonstrated high MOI values, failing to capture approximately 69–73% of early-stage (Stage I–II) cases. Conversely, age-only minimal models appeared highly performant, structurally capturing approximately 95% of the cohort due to broad inclusiveness. **Conclusions:** In retrospective eligibility simulations, smoking-based screening models were associated with high missed opportunity rates for Stage I–II cases, highlighting a mismatch between simplified Western risk models and the multidimensional reality of lung cancer epidemiology in this cohort. These findings strongly support the need to move toward multivariable risk prediction models and population-adapted screening strategies that integrate non-traditional risk factors to better align screening eligibility with the true distribution of disease in real-world settings.

## 1. Introduction

Lung cancer remains the most frequently diagnosed malignant neoplasm and the leading cause of cancer-related mortality globally, representing a massive and complex public health burden [1,2]. Despite paradigm-shifting advancements in targeted therapies and immune checkpoint inhibitors over the last decade, the overall prognosis for lung cancer remains heavily dictated by the stage of the disease at the time of diagnosis. When detected in its earliest, asymptomatic stages, surgical resection and stereotactic body radiation offer high probabilities of curative intent; however, the insidious nature of the disease means that the vast majority of patients remain undiagnosed until they develop debilitating systemic or respiratory symptoms [3,4]. Consequently, early detection through organized screening programs is universally recognized as the most effective strategy to achieve a meaningful reduction in population-level mortality. Yet, the translation of large-scale, controlled screening trials such as the US National Lung Screening Trial (NLST) or the European Dutch–Belgian Lung Cancer Screening Trial (NELSON) into real-world, scalable public health policies is fraught with epidemiological, economic, and structural complexities [5,6]. Despite increasing European-level support for LDCT-based lung cancer screening, implementation remains heterogeneous across countries, ranging from established or emerging organized programs to pilot initiatives and healthcare systems in which national population-based screening pathways have yet to be established.

Nations in Central and Eastern Europe (CEE), such as Romania, Poland, Hungary, and Bulgaria [7,8], share a distinct epidemiological burden of lung cancer [7,9]. Romania represents a particularly challenging scenario, where lung cancer claims over 10,000 lives annually, and the vast majority of patients (approximately 88–89%) present with advanced-stage disease (Stages III–IV), severely limiting [10]. A defining and severe feature of the Romanian clinical landscape is the alarming rate of late-stage presentations. Real-world data indicates that over 60% of Romanian patients are diagnosed at Stage IV, with an additional 43% presenting at Stage III, drastically limiting therapeutic options to palliative systemic therapies and driving an exceptionally high mortality rate [11]. Furthermore, the country currently lacks a comprehensive, centralized national lung cancer registry and does not yet have a formal, nationwide population-based screening program, resulting in massive diagnostic gaps and fragmented patient pathways [12,13].

The absence of an organized screening infrastructure means that current clinical datasets in Romania predominantly reflect a symptomatic cohort rather than an asymptomatic screening population. Diagnoses are overwhelmingly made *post hoc*, often triggered incidentally during acute hospital admissions or advanced disease presentations [14]. Analyzing such cohorts introduces a fundamental selection bias; however, it also provides a crucial real-world window into the systemic failures of early detection. When standard international screening guidelines which rely almost exclusively on strict age parameters and cumulative smoking exposure (pack-years) are retrospectively applied to these cohorts, they reveal a massive MOI. Strict adherence to these guideline-based Western models often results in the artificial misclassification of highly vulnerable individuals as “non-eligible” for screening [15,16].

This mismatch between simplified risk models and the multidimensional reality of lung cancer epidemiology in Romania may be influenced by several regional particularities. First, standard smoking-based criteria fail to capture patients with non-smoking-related lung cancers, a demographic that is increasingly prominent. Recent genomic profiling of Romanian non-small cell lung cancer (NSCLC) cohorts has uncovered a high prevalence of actionable driver mutations, notably epidermal growth factor receptor (EGFR) mutations occurring predominantly in non-smoking females, and Kirsten rat sarcoma viral oncogene homolog (KRAS) p.G12C alterations [17,18]. Relying solely on pack-year metrics systematically excludes these genetically susceptible populations from early diagnostic workflows.

Second, the Romanian landscape is heavily influenced by non-traditional and environmental risk determinants that standard models ignore. Occupational and residential exposures, such as high indoor radon concentrations in regions like Transylvania, and heavy particulate matter (PM) air pollution in industrialized zones, independently drive tumorigenesis [19,20]. Furthermore, clustering analyses have identified specific high-risk phenotypes, such as “rural extreme smokers” who exhibit severe, often undiagnosed chronic respiratory diseases (e.g., chronic obstructive pulmonary disease (COPD) and pulmonary fibrosis) alongside lung cancer [14,21].

Perhaps the most critical barrier to scalable screening in Romania is the tuberculosis (TB) confounder. Romania possesses one of the highest incidences of TB in the European Union. Consequently, a large proportion of the adult population harbors post-inflammatory fibronodular lesions, tuberculomas, and benign granulomas [22]. On standard low-dose computed tomography (LDCT), these benign sequelae radiologically mimic early-stage malignancies. If a screening program utilizes standard spatial features (like nodule diameter) without adjusting for this regional epidemiological noise, it generates an unacceptable rate of false-positive results, leading to unnecessary, invasive, and costly surgical biopsies for benign diseases [23].

To overcome these profound contextual mismatches, it is evident that a simple “copy–paste” of Western screening protocols will not suffice in the CEE region. There is an urgent need to transition toward multivariable risk prediction models and population-adapted screening strategies. Drawing on innovations from neighboring countries such as Poland’s pioneering use of serum metabolomic and microRNA (miRNA) signatures as biological “gatekeepers” to filter out false positives, and Hungary’s adoption of strict volumetric doubling time (VDT) criteria Romania must define a scalable pathway that integrates both radiological precision and biological triage [6,24,25]. To address these regional gaps and account for the high rate of missing data typical of routine clinical practice in CEE, we developed a dual-cohort methodological approach. This design allows us to preserve population-level representativeness while conducting detailed screening eligibility simulations in a granular, complete-case subset, thereby bridging the gap between theoretical guidelines and real-world data constraints.

We hypothesized that standard Western, smoking-based screening criteria systematically under-select high-risk individuals in Central and Eastern Europe due to regional epidemiological disparities. To address this, the primary objective of this study—conducted within the framework of the SOLACE project (https://europeanlung.org/solace/ (accessed on 28 January 2026))—was to evaluate theoretical eligibility and calculate the missed opportunity index (MOI) of established guidelines (USPSTF, NCCN, and ERS) in a retrospective Romanian tertiary cohort (n = 3764). Through this evaluation, we aim to provide the real-world evidence necessary to design population-adapted screening strategies that integrate regional environmental, genetic, and infectious confounders.

## 2. Materials and Methods

### 2.1. Study Design and Setting

This study is a retrospective, observational analysis conducted using real-world clinical data from lung cancer patients treated at the Oncological Institute of Bucharest (IOB), Romania. The objective was to characterize diagnostic patterns, stage distribution, and healthcare pathways, and to derive data-driven insights for the potential scaling of lung cancer screening programs in a CEE context.

The study was designed as a dual-cohort analysis based on data completeness: Set A (full cohort), comprising all identified lung cancer patients within the study period, and Set B (complete-case subset), including patients from Set A for whom quasi-complete clinical, diagnostic, and temporal data were available. From a clinical and diagnostic standpoint, a complete case was defined as a patient with documented lung cancer histology, recorded clinical TNM staging, and receipt of at least one established oncologic treatment (surgery, chemotherapy, radiotherapy, immune checkpoint inhibitors, or targeted therapy). These criteria for a complete case were defined before data extraction. This dual-cohort framework enabled the separation of population-level analyses (Set A), preserving external validity, from more granular analyses of diagnostic pathways and timing (Set B), providing internal analytical consistency. Importantly, this design allowed for the explicit assessment of missing data patterns and potential selection bias associated with complete-case analysis.

### 2.2. Study Population

The study included all consecutive patients diagnosed with lung cancer and managed at IOB within the 1990 to 2023 timeframe. Patients were systematically discussed within the institutional multidisciplinary team (MDT), where therapeutic strategy, including assessment of resectability when applicable, was established through multidisciplinary evaluation. Inclusion criteria were: (1) histologically or cytologically confirmed lung cancer and (2) availability of at least minimal demographic and diagnostic data. Patients with incomplete or missing data were retained in Set A to preserve representativeness. No exclusion was performed based on disease stage, histology, or treatment intent.

Set B was defined post hoc as the subset of patients meeting the complete-case criteria specified above, namely documented lung cancer histology, recorded clinical TNM staging, and documentation of at least one established oncologic treatment. No randomization or predefined selection criteria were applied; inclusion in Set B depended solely on data completeness.

The use of two analytical cohorts was intentional and methodologically justified. Set A, comprising all identified patients, was used to preserve external validity and accurately reflect the real-world distribution of disease stage and clinical characteristics. Set B, a complete-case subset, was used for analyses requiring more detailed clinical and temporal data, such as diagnostic pathway evaluation and screening simulation. Comparative analyses between Set B and patients with incomplete data were performed to assess documentation-related selection bias. Because inclusion in Set B depended on data availability and differences between the subsets were observed for several relevant characteristics, Set B was not assumed to be representative of the full cohort. Rather, it was considered an internally consistent subset for analyses requiring greater clinical and exposure-data completeness.

### 2.3. Data Collection and Variables

Data were extracted from institutional clinical records, including electronic medical records, pathology reports, and imaging documentation. The collected variables were categorized according to their availability across the full cohort (Set A) and the complete-case subset (Set B), distinguishing routinely available data from variables obtainable only in patients with more complete clinical documentation (Table 1).

### 2.4. Statistical Analysis

All statistical analyses were performed using standard statistical software R 4.5.2. A two-cohort analytical framework was applied, with Set A used for population-level estimates and Set B for analyses requiring higher data completeness.

#### 2.4.1. Descriptive Analysis

Descriptive statistics were used to summarize patient characteristics across both datasets. Continuous variables (e.g., age) were reported as mean ± standard deviation (SD) or median with interquartile range (IQR), depending on distribution. Categorical variables (e.g., sex, histological subtype, stage group) were presented as counts and percentages. All proportions were calculated using explicit denominators (n/N) to account for missing data, particularly within Set A.

#### 2.4.2. Completeness and Selection Bias Analysis

To evaluate the impact of missing data, a complete-case analysis framework was applied. Patients included in Set B (complete cases) were compared with those excluded due to incomplete data (Set A incomplete subset).

Comparisons were performed for key baseline variables, including age (continuous), sex (categorical), histological subtype, and stage at diagnosis. Continuous variables were compared using Student’s *t*-test or Mann–Whitney U test, as appropriate. Categorical variables were compared using χ^2^ test or Fisher’s exact test.

This analysis aimed to characterize the mechanism of missingness (missing completely at random, missing at random, or not at random) and to assess the presence and magnitude of potential selection bias.

#### 2.4.3. Stage Distribution and Disease Burden Analysis

For analytical clarity, stages were grouped into early-stage disease (Stages I–II) and advanced-stage disease (Stages III–IV). Stage distribution was calculated primarily in Set A to preserve external validity. Results were expressed as the proportion of early-stage and advanced-stage cases, as well as the proportion of Stage IV disease. A sensitivity analysis using Set B was conducted to evaluate the robustness of these estimates in a cohort with higher data completeness.

#### 2.4.4. Screening Eligibility and Simulation Analysis

Screening eligibility was evaluated using a two-step approach. A minimal model was applied to Set A, based primarily on age criteria, to maximize sample size and reflect broad population-level screening strategies. An extended model was applied to Set B, incorporating additional risk factors such as smoking status (when available), approximating established screening criteria [National Comprehensive Cancer Network (NCCN), United States Preventive Services Task Force (USPSTF), European Respiratory Society (ERS)]. Table 2 summarizes the screening eligibility criteria recommended by the NCCN, ERS, and USPSTF against which our analysis was conducted. These analyses were designed as retrospective counterfactual eligibility simulations among patients with established lung cancer and were not intended to estimate screening sensitivity, diagnostic performance, or effectiveness in an asymptomatic screening population.

The age-only scenarios were evaluated solely as theoretical comparators to examine the effect of progressively adding smoking-related eligibility restrictions and were not intended to represent proposed screening strategies. A missed opportunity index (MOI) was calculated to quantify the proportion of early-stage lung cancer cases that would not have met the evaluated screening eligibility criteria. For each screening scenario, MOI was defined as:


$$MOI=\frac{\mathrm{Stage}\;I\text{–}\mathrm{II}\;\mathrm{patients}\;\mathrm{classified}\;\mathrm{as}\;\mathrm{not}\;\mathrm{eligible}}{\mathrm{all}\;\mathrm{Stage}\;I\text{–}\mathrm{II}\;\mathrm{patients}\;\mathrm{in}\;\mathrm{the}\;\mathrm{relevant}\;\mathrm{eligibility}\;\mathrm{denominator}}\times 100$$

MOI is a study-specific descriptive metric and should not be interpreted as a validated measure of screening sensitivity or specificity; in particular, specificity cannot be estimated from this case-only cohort. Accordingly, a higher MOI indicates a greater proportion of retrospectively identified early-stage cases that would have been classified as non-eligible under the corresponding screening scenario.

To estimate the potential impact of screening, a simplified stage-shift model was applied. Based on evidence from landmark screening trials, a conservative proportion of advanced-stage cases was hypothetically reclassified as early-stage. The resulting change in stage distribution was used to estimate the potential increase in potentially curable cases.

#### 2.4.5. Hypothetical Screening Impact

A simplified stage-shift model was applied to estimate the potential impact of screening. Based on published literature [26,27,28,29,30], a conservative proportion of advanced-stage cases was hypothetically reclassified as early-stage. The resulting change in stage distribution was calculated to estimate the potential increase in potentially curable cases.

#### 2.4.6. Sensitivity Analysis

To explore the potential magnitude of a hypothetical screening-associated stage shift while accounting for uncertainty arising from missing stage information, two scenario-based simulations were performed. In the optimistic scenario, patients with missing stage were assigned to Stages I–II or Stages III–IV according to the observed proportional distribution among patients with available stage information. In the pessimistic scenario, missing-stage cases were assumed to have a predominantly advanced-stage distribution, with 10% assigned to Stages I–II and 90% to Stages III–IV. Following reconstruction of the baseline stage distribution under each scenario, a hypothetical stage shift was applied by reclassifying 10% of Stages III–IV cases as Stages I–II in the optimistic scenario and 5% in the pessimistic scenario. These shift proportions were selected as conservative scenario assumptions informed by previously reported screening-associated stage-shift estimates [26,27,28,29,30] and were not estimated from the present cohort. The simulations therefore represent hypothetical sensitivity analyses rather than predictions of the causal effect of screening implementation.

These analyses provided a plausible range for key outcomes (e.g., late-stage proportion, screening impact).

To assess the robustness of guideline-based screening eligibility estimates to missing smoking-related information, additional sensitivity analyses were performed using alternative missing-data assumptions. The primary conservative analysis classifies indeterminate eligibility due to missing essential smoking-related information as not eligible. The available-case analysis excludes indeterminate patients from the denominator. The worst-case bound assigns all indeterminate cases as not eligible. The best-case bound assigns indeterminate cases as eligible only when observed non-missing guideline components do not already establish ineligibility. USPSTF is implemented approximately because exact years since smoking cessation are unavailable; documented former smokers are therefore accepted for the smoking-status component. NCCN uses the available ≥20 pack-year branch because validated total smoking duration is unavailable. For each sensitivity scenario, MOI was calculated using the same definition as in the primary analysis (§2.5.4), as the proportion of Stage I–II patients classified as not eligible among all Stage I–II patients included in the corresponding scenario-specific eligibility denominator. Eligibility was recalculated independently for each scenario, and patients with insufficient information to determine eligibility under a given scenario were excluded from the corresponding denominator.

## 3. Results

### 3.1. Data Completeness and Cohort Definition

Data completeness varied substantially across variables, with near-complete demographic data but high levels of missingness for molecular and exposure-related variables (Table 3). The analysis of data completeness highlights a heterogeneous distribution of missingness across variable domains. Demographic variables demonstrated excellent completeness, with age and sex available for all patients (100%), supporting the robustness of population-level descriptions. In contrast, clinical tumor characterization showed only moderate completeness, with stage available in approximately one-quarter of the cohort (23.6%) and histological subtype in just over half (57.4%), reflecting limitations in routine documentation or data extraction. More importantly, variables directly relevant to screening strategies exhibited substantial missingness. Smoking status, a key determinant of eligibility in current screening guidelines, was unavailable in approximately 70% of cases. Similarly, molecular biomarkers such as EGFR, ALK, and PD-L1 were missing in roughly 75–80% of patients, indicating limited access or inconsistent testing in real-world practice.

This pattern of missingness underscores a critical gap between theoretical screening frameworks and their practical implementation in data-constrained settings, and provides a strong methodological justification for the dual-cohort design employed in this study.

**Figure 1:**
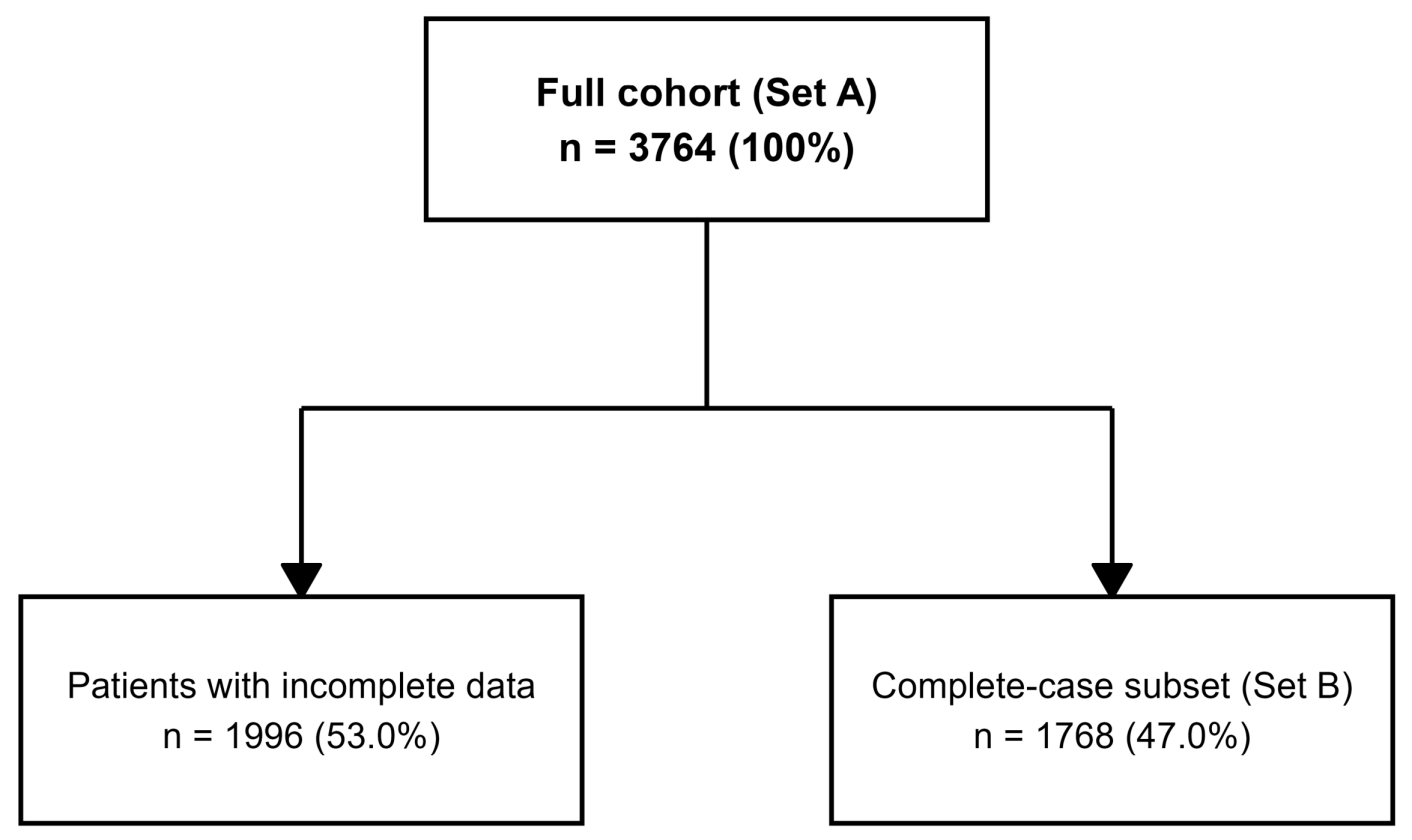
Flow diagram illustrating cohort definition and data completeness.

The derivation of analytical cohorts is illustrated in Figure 1. The cohort construction process highlights the substantial impact of data completeness on the analytical framework. Of the 3764 patients included in the full cohort (Set A), slightly more than half (53.0%, n = 1996) had incomplete data, while 47.0% constituted the complete-case subset (Set, n = 1768).

This distribution underscores the high degree of missingness affecting key clinical and exposure variables, necessitating a dual-cohort design. Comparative analyses between Set B and patients with incomplete data were performed to assess potential documentation-related selection bias. Because inclusion in Set B depended on data availability and differences between the subsets were observed for several relevant characteristics, Set B was not assumed to be representative of the full cohort. Rather, it was considered an internally consistent subset for analyses requiring greater clinical and exposure-data completeness. The relatively large proportion of incomplete cases further supports the presence of systematic limitations in data collection within routine clinical practice.

Patients included in Set B differed from those with incomplete data in several baseline characteristics (Table 4). Comparative analysis between complete and incomplete cases revealed evidence of non-random missingness. Patients included in Set B were slightly younger than those with incomplete data (66.3 ± 8.9 vs. 66.9 ± 9.4 years, *p* < 0.001), while sex distribution was not significantly different (*p* = 0.092). Marked discrepancies were observed for tumor-related variables. Staging information was available for a significantly higher proportion of patients in Set B compared to the incomplete subset (45.6% vs. 23.6%, with staging missing in 54.4% of Set B and 76.4% of the incomplete subset, respectively). Among patients with available staging, the proportion of early-stage disease did not differ significantly between Set B and Set A (8.1% vs. 4.2% of staged patients, overall *p* = 0.728), indicating that although staging documentation was systematically limited, the underlying stage distribution among staged cases remained comparable.

Histological data showed a similar pattern. While major subtypes [adenocarcinoma, squamous cell carcinoma (SCC), small-cell lung cancer (SCLC)] were more frequently identified in Set B, the incomplete cohort exhibited a substantially higher proportion of both unspecified (“other”) and missing histologies. Notably, missing histology accounted for 42.6% of incomplete cases, whereas no missing values were present in Set B.

These findings confirm that missingness is not random but structurally associated with diagnostic completeness, supporting the use of a dual-cohort design and highlighting potential biases in complete-case analyses.

### 3.2. Population Characteristics and Risk Profile

Baseline demographic and exposure-related characteristics of the study population are summarized in Table 5.

The demographic structure of the cohort was comparable between Set A and Set B, with similar age distributions and a predominance of older patients (patients aged ≥ 60 years accounting for 80.5% of Set A and 80.1% of Set B). Men predominated in both cohorts, representing 69.5% of Set A and 70.9% of Set B.

In contrast, risk factor variables showed marked differences in completeness and distribution. Smoking-related data were substantially more available in Set B, with smoking status missing for 71.4% of Set A and 50.2% of Set B. Cumulative tobacco exposure was even less complete, with pack-year information missing for 82.8% and 70.1% of Set A and Set B, respectively. Consequently, when percentages were calculated using the full cohort denominators, the reported proportions were higher in Set B for current smokers (34.0% vs. 19.3%), former smokers (11.7% vs. 6.8%), and patients with ≥30 pack-years of exposure (22.1% vs. 12.9%). These differences primarily reflect the greater availability of smoking-related information in the complete-case subset rather than direct evidence of differences in the underlying exposure distribution.

These findings indicate that while demographic characteristics are robust across cohorts, risk factor data are subject to significant missingness and potential selection bias. This discrepancy has important implications for screening eligibility analyses, which rely heavily on accurate smoking exposure assessment.

Row-wise comparisons between Set A and Set B revealed consistent and statistically significant differences in smoking status across multiple strata (Table 6). Across all age categories, the proportion of current smokers was similar in Set A and Set B: 86.8% versus 89.7% among patients aged < 50 years (*p* = 1.000), 78.9% versus 79.1% at 50–59 years (*p* = 0.974), 72.9% versus 72.8% at 60–69 years (*p* = 0.994), and 72.2% versus 73.0% at ≥70 years (*p* = 0.809). None of these between-cohort differences was statistically significant.

Sex-stratified analysis demonstrated a similar pattern. Among men, current smokers represented 73.3% of evaluable patients in Set A and 74.3% in Set B (*p* = 0.659), while among women the corresponding proportions were 77.0% and 75.3% (*p* = 0.692). Thus, no significant between-cohort differences in the current-versus-former smoking distribution were observed by sex.

Similarly, cumulative tobacco exposure (pack-years) showed minimal variation between the two cohorts. The proportion of patients with ≥30 pack-years was 42.3% versus 40.0% among those aged <50 years (*p* = 0.875), 63.6% versus 62.5% at 50–59 years (*p* = 0.872), 80.6% versus 81.2% at 60–69 years (*p* = 0.870), and 76.7% versus 74.2% at ≥70 years (*p* = 0.547), for Set A and Set B, respectively. Likewise, no significant between-cohort differences were observed among men or women.

Taken together, these findings suggest that the complete-case subset contains substantially more complete smoking and pack-year information, while the distributions of smoking status and cumulative tobacco exposure among patients with available data remain highly consistent between Set A and Set B across age and sex strata. Thus, the principal difference between the cohorts appears to concern data completeness rather than the observed distribution of tobacco-exposure characteristics among evaluable patients. This distinction has important implications for screening eligibility analyses that rely on smoking-based criteria.

### 3.3. Tumor Characteristics and Disease Burden

Associations between histological subtype and demographic, exposure, clinical, and geographic variables are presented in Table 7. Histological subtype was significantly associated with several key variables. Strong associations were observed for sex, age, smoking status, cumulative tobacco exposure, and staging categories (all *p* < 0.05), whereas no significant association was identified for living environment.

Adenocarcinoma was significantly more frequent among female patients and younger age groups, while squamous cell carcinoma (SCC) was more prevalent among males, older patients, and individuals with higher cumulative tobacco exposure. These associations were confirmed by both global and category-specific analyses.

Smoking-related variables showed a particularly robust relationship with histology. Current smoking and heavy exposure were significantly associated with increased SCC prevalence and decreased adenocarcinoma proportion, whereas never smoking and lower exposure showed the opposite pattern.

Stage at diagnosis was also associated with histological subtype. Adenocarcinoma was more frequent in Stage I–II than in Stage III–IV disease (54.5% vs. 45.1%; *p* = 0.040), whereas SCLC was substantially more frequent in Stage III–IV than in Stage I–II disease (13.0% vs. 3.5%; *p* = 0.001). The overall association between staging category and histological distribution was significant (*p* = 0.004).

In contrast, geographic variation was limited. Histological distributions were similar between urban and rural patients, with no significant overall association between living environment and histological subtype (*p* = 0.560).

In contrast, other histologies showed a relatively stable distribution across most variables, whereas SCLC demonstrated significant variation according to smoking status and staging category. Overall, these findings reinforce the strong etiological link between tobacco exposure and histological subtype, while highlighting the additional influence of demographic and disease-stage factors.

**Figure 2:**
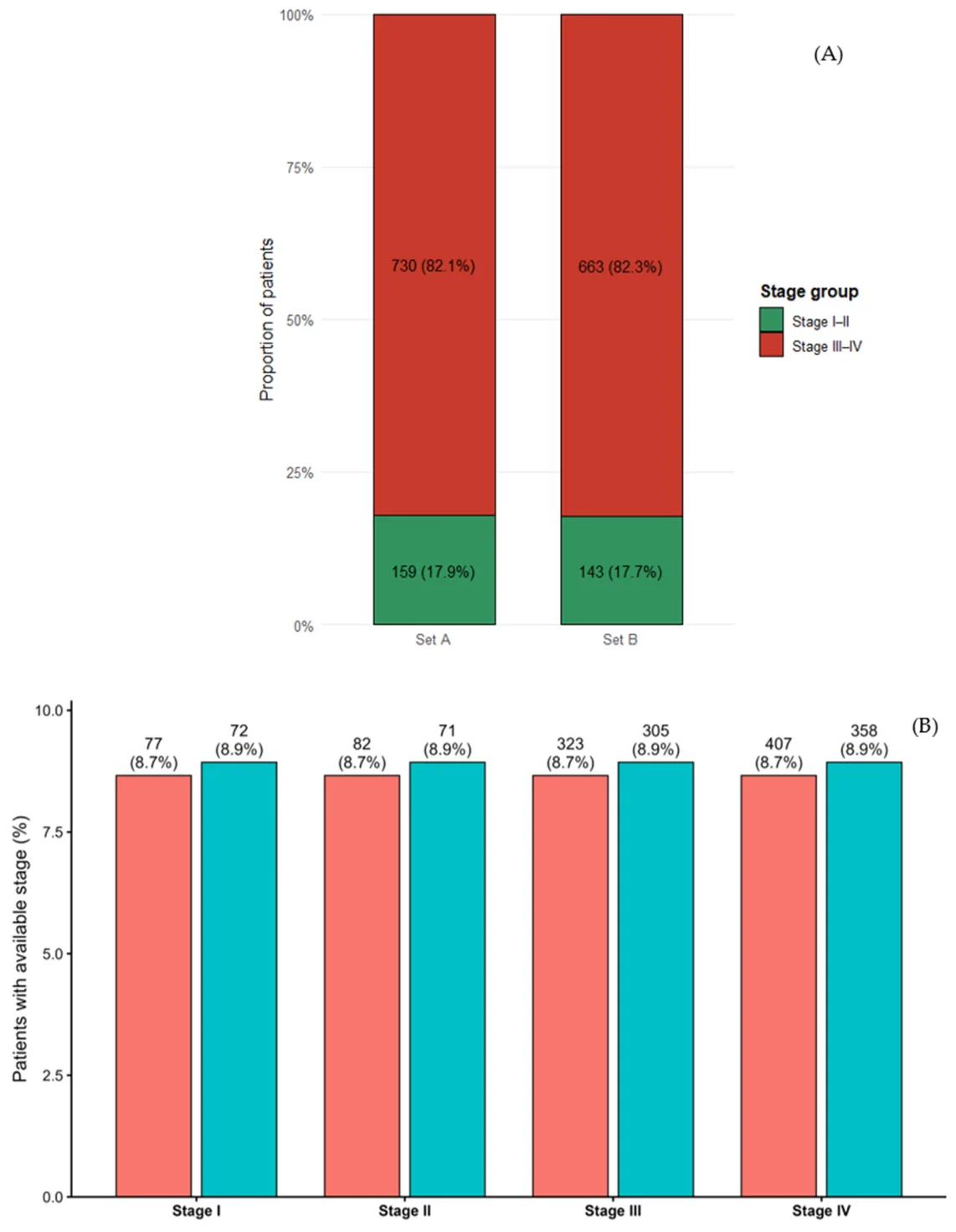
(**A**) Cumulated distribution of early-stage disease (Stages I–II) and advanced-stage disease (Stages III–IV) and (**B**) separated distribution of all stages of lung cancer in the full cohort (Set A) and the complete-case subset (Set B).

Most patients were diagnosed at advanced stages (Figure 2). Stage distribution was comparable between Set A and Set B. In both datasets, most patients presented with advanced disease (Stages III–IV), accounting for approximately 82% of cases with available stage, while early-stage disease (Stages I–II) accounted for approximately 18%.

No significant difference in stage distribution was observed between the full cohort (Set A) and the complete-case subset (Set B) (17.9% vs. 17.7% for Stages I–II and 82.1% vs. 82.3% for Stages III–IV, respectively), supporting the absence of stage-related selection bias. This supports the validity of using Set B for more detailed analyses, despite its restriction to patients with more complete data.

Overall, the predominance of advanced-stage disease highlights the critical need for earlier detection strategies and supports the implementation of structured lung cancer screening programs in this population.

When the four individual TNM stages were examined separately (Figure 2B), their distributions were also highly similar between Set A and Set B. Stage I represented 8.7% and 8.9% of patients with available stage, respectively; Stage II, 8.7% and 8.9%; Stage III, 8.7% and 8.9%; and Stage IV, 8.7% and 8.9%.

Thus, Figure 2 primarily demonstrates the stability of stage distribution between the full cohort and the complete-case subset. In both cohorts, Stage III–IV disease accounted for more than four-fifths of patients with available staging information, whereas Stage I–II disease represented fewer than one-fifth. These findings further support the representativeness of Set B with respect to disease-stage distribution and reinforce the substantial burden of advanced-stage disease in the study population.

Associations between TNM stage and demographic, exposure, and geographic variables are summarized in Table 8. Significant associations between TNM stage distribution and sex were observed (overall *p* < 0.001), whereas age, smoking status, cumulative tobacco exposure, and living environment did not demonstrate statistically significant overall associations with stage at diagnosis.

Women had a significantly higher proportion of Stage I disease than men (15.6% vs. 6.4%, *p* < 0.001), whereas Stage III disease was more frequent among men (40.2% vs. 31.7%, *p* = 0.026). Stage II and Stage IV proportions did not differ significantly by sex. No significant overall association was observed between age category and stage distribution (*p* = 0.316). Although the observed proportions varied across age groups, none of the stage-specific comparisons reached statistical significance.

Similarly, stage distribution did not differ significantly between current and former smokers (overall *p* = 0.875) or between patients with <30 and ≥30 pack-years of cumulative tobacco exposure (overall *p* = 0.946). None of the corresponding stage-specific comparisons was statistically significant. Geographic differences were also not evident: stage distribution was comparable between urban and rural patients (overall *p* = 0.548), with no significant differences for any individual stage.

Importantly, Stage IV proportions remained consistently high across all categories and did not significantly differ between groups, indicating that metastatic presentation is a pervasive feature of this cohort. Overall, these findings highlight that while sex was significantly associated with overall TNM stage distribution, age, smoking status, cumulative tobacco exposure, and living environment were not. The higher proportion of Stage I disease among women and Stage III disease among men suggests a sex-related difference in stage at presentation, whereas the consistently high Stage IV burden across strata reinforces the need for targeted early detection strategies.

**Figure 3:**
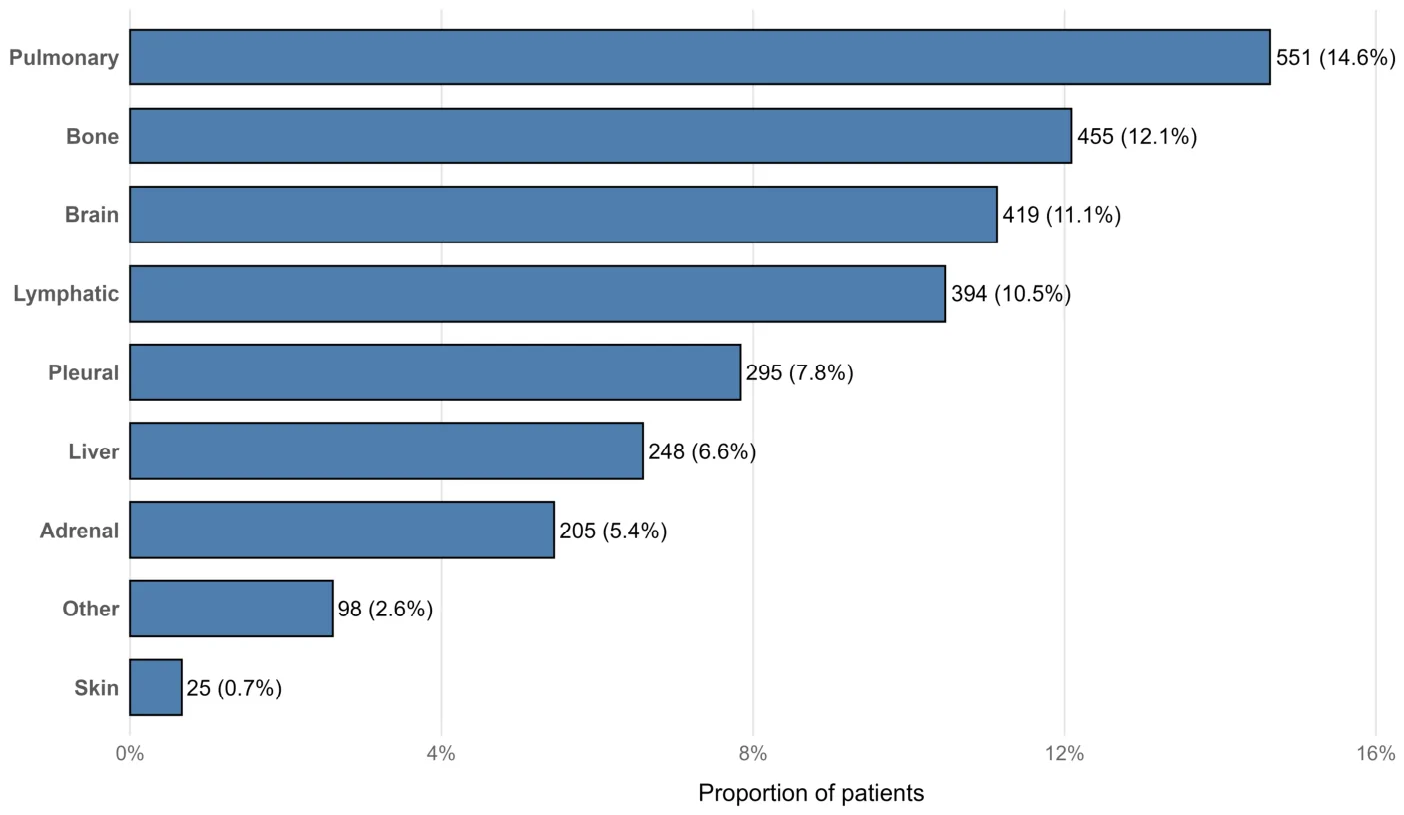
Distribution of metastatic sites in the full cohort (Set A). Bars show the proportion of patients with documented involvement of each metastatic site.

The distribution of metastatic sites in the full cohort is shown in Figure 3. The distribution of metastatic sites revealed a heterogeneous but clinically coherent pattern. Pulmonary involvement was the most frequent metastatic presentation, affecting 551 patients (14.6%), followed by bone metastases in 455 (12.1%) and brain metastases in 419 (11.1%). Lymphatic involvement was documented in 394 patients (10.5%), while pleural involvement occurred in 295 (7.8%), reflecting advanced locoregional and systemic spread.

Visceral metastases such as liver and adrenal involvement were less frequent but remained clinically significant affecting 248 (6.6%) and 205 (5.4%) patients, respectively, while skin and other rare sites were uncommon (25 [0.7%] and 98 [2.6%], respectively). This distribution highlights the predominance of thoracic and skeletal dissemination pathways in this population.

Importantly, the high overall burden of metastatic disease across multiple sites reinforces the observation that most patients present with advanced-stage disease, further supporting the need for earlier detection through structured screening strategies.

### 3.4. Molecular Profile

Molecular testing was not uniformly performed across the cohort; therefore, analyses were restricted to patients with available results for each biomarker, and percentages were calculated using biomarker-specific denominators. These results should be interpreted with caution due to potential selection bias in testing practices. Accordingly, the reported molecular alteration frequencies should not be interpreted as prevalence estimates for the entire cohort or the Romanian lung cancer population, and the observed associations may reflect both underlying biological patterns and selective testing practices. Associations between molecular alterations and clinical, demographic, and exposure variables are presented in (Table 9).

Molecular alterations demonstrated significant associations with several demographic and exposure variables. EGFR mutations were significantly more frequent in males (34.0% vs. 16.6%; *p* < 0.001) and in patients with <30 pack-years of cumulative tobacco exposure (25.4% vs. 9.8% for ≥30 pack-years; *p* = 0.005). EGFR positivity also differed significantly by living environment, being higher in urban than rural patients (24.8% vs. 17.6%; *p* = 0.024), whereas its association with age did not reach statistical significance (*p* = 0.070). ALK rearrangements showed a similar but less pronounced pattern, with significantly higher positivity in females than males (12.4% vs. 4.7%; *p* < 0.001) and significant variation across age categories (*p* = 0.002), including the highest proportion among patients aged < 50 years (25.6%). In contrast, PD-L1 expression did not demonstrate consistent associations with demographic or exposure variables, and no significant association with staging category was observed (*p* = 0.356).

No statistically significant differences in molecular alterations were identified between urban and rural populations, suggesting that geographic disparities observed in clinical presentation are unlikely to be driven by underlying tumor biology. Overall, these findings highlight distinct epidemiological patterns of targetable molecular alterations, supporting the inclusion of non-smoking and female populations in screening and diagnostic strategies.

### 3.5. Geographic Distribution and Disparities

**Figure 4:**
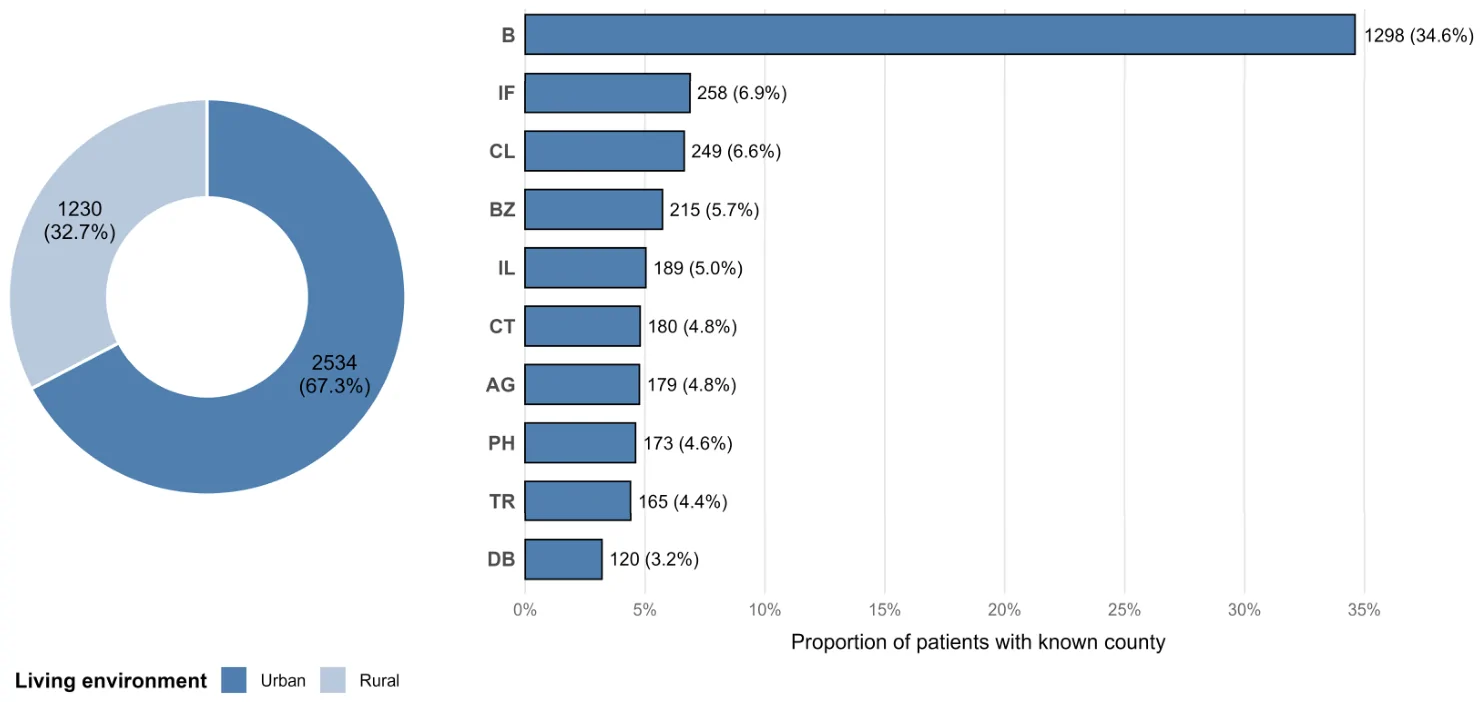
Distribution of patients by living environment (**left**)—rural and urban—and by county of origin (**right**) in the full cohort (Set A). The figure displays the 10 counties contributing the highest proportion of cases (B—Bucharest and the counties: IF—Ilfov, CL—Calarasi, BZ—Buzău, IL—Ialomita, CT—Constanta, AG—Argeș, PH—Prahova, TR—Teleorman, DB—Dambovita).

The distribution of patients according to living environment showed that the majority of cases originated from urban areas, accounting for 67.3% (n = 2534) of the cohort (Figure 4). Rural patients accounted for the remaining 32.7% (n = 1230).

Among patients with known county of origin, Bucharest represented the largest geographic contribution, accounting for 34.6% (n = 1298) of cases. The next most frequently represented counties were Ilfov (6.9%; n = 258), Călărași (6.6%; n = 249), Buzău (5.7%; n = 215), Ialomița (5.0%; n = 189), Constanța (4.8%; n = 180), Argeș (4.8%; n = 179), Prahova (4.6%; n = 173), Teleorman (4.4%; n = 165), and Dâmbovița (3.2%; n = 120).

### 3.6. Screening Eligibility and Missed Opportunities

#### 3.6.1. Screening Eligibility—Minimal Model

Screening eligibility based on the minimal model (age criteria only) was evaluated in the full cohort (Set A). Eligibility was calculated using age information, whereas stage distributions among eligible patients were calculated only for those with available staging classification. Screening eligibility based on age-only criteria was high across all tested scenarios (Table 10). Using the most inclusive threshold (≥50 years), 95.38% of patients with available stage information were classified as eligible, while the ≥55 years criterion reduced eligibility to 89.77%. Restricting eligibility to the 50–80-year interval resulted in an intermediate proportion of 89.05%.

Across all scenarios, the vast majority of eligible patients with available staging information were diagnosed at advanced stages (Stages III–IV), accounting for approximately 81–82% of cases, whereas early-stage disease (Stages I–II) represented only about 18–19%. Importantly, this distribution remained remarkably stable across different age thresholds.

These findings indicate that, as a theoretical comparator, broad age-only eligibility retrospectively classifies a large proportion of patients in this cohort as eligible; however, most eligible patients with available staging information had advanced-stage disease at diagnosis. This highlights a substantial gap between theoretical screening eligibility and effective early detection, supporting the need for additional risk stratification and earlier implementation of screening strategies.

#### 3.6.2. Screening Eligibility—Extended Models

The application of established guideline-based screening criteria (as extended models) demonstrates several consistent and clinically meaningful patterns (Table 11). First, the proportion of patients meeting eligibility criteria remains limited across all evaluated models, ranging from 22.40% under ERS criteria to 25.62% under NCCN criteria, with USPSTF identifying 24.38% as eligible. This finding indicates that most patients within this cohort would not have qualified for screening according to current recommendations, thereby highlighting a substantial discrepancy between real-world case presentation and populations defined as high-risk by existing screening frameworks.

Second, among the evaluated models, the NCCN criteria identify the largest proportion of eligible individuals, followed closely by USPSTF, whereas ERS recommendations appear the most restrictive regarding overall eligibility. The absolute differences between models were relatively modest, with eligibility ranging by only 3.22% points between NCCN and ERS.

Third, the distribution of disease stage among eligible patients remains highly consistent across all screening models. Early-stage disease (Stages I–II) accounts for approximately 18.48–18.78% of cases, whereas advanced-stage disease (Stages III–IV) comprises approximately 81.22–81.52%. This uniformity suggests that, despite variations in inclusiveness, current eligibility criteria do not substantially improve the detection of early-stage disease within this dataset. Notably, even within populations theoretically enriched for risk, the predominance of advanced-stage diagnoses persists.

Fourth, the absolute number of early-stage cases identified within the eligible population remains modest across all models, with 43 cases detected under USPSTF criteria, 45 under NCCN, and 39 under ERS. This finding underscores a critical limitation of current screening strategies, namely their limited capacity to capture a substantial proportion of patients at a stage amenable to curative intervention.

Finally, these observations support the hypothesis that risk stratification approaches based exclusively on age and smoking exposure may be insufficient for the effective identification of early-stage lung cancer in real-world populations. Consequently, these results reinforce the rationale for advancing toward more comprehensive risk assessment strategies, including the incorporation of multivariable prediction models [e.g., prostate, lung, colorectal and ovarian cancer screening trial model 2012 (PLCOm2012)], integration of additional risk determinants such as genetic susceptibility, environmental exposures, and comorbid conditions, as well as the potential implementation of biomarker-driven screening approaches.

#### 3.6.3. Missed Opportunity Index

**Figure 5:**
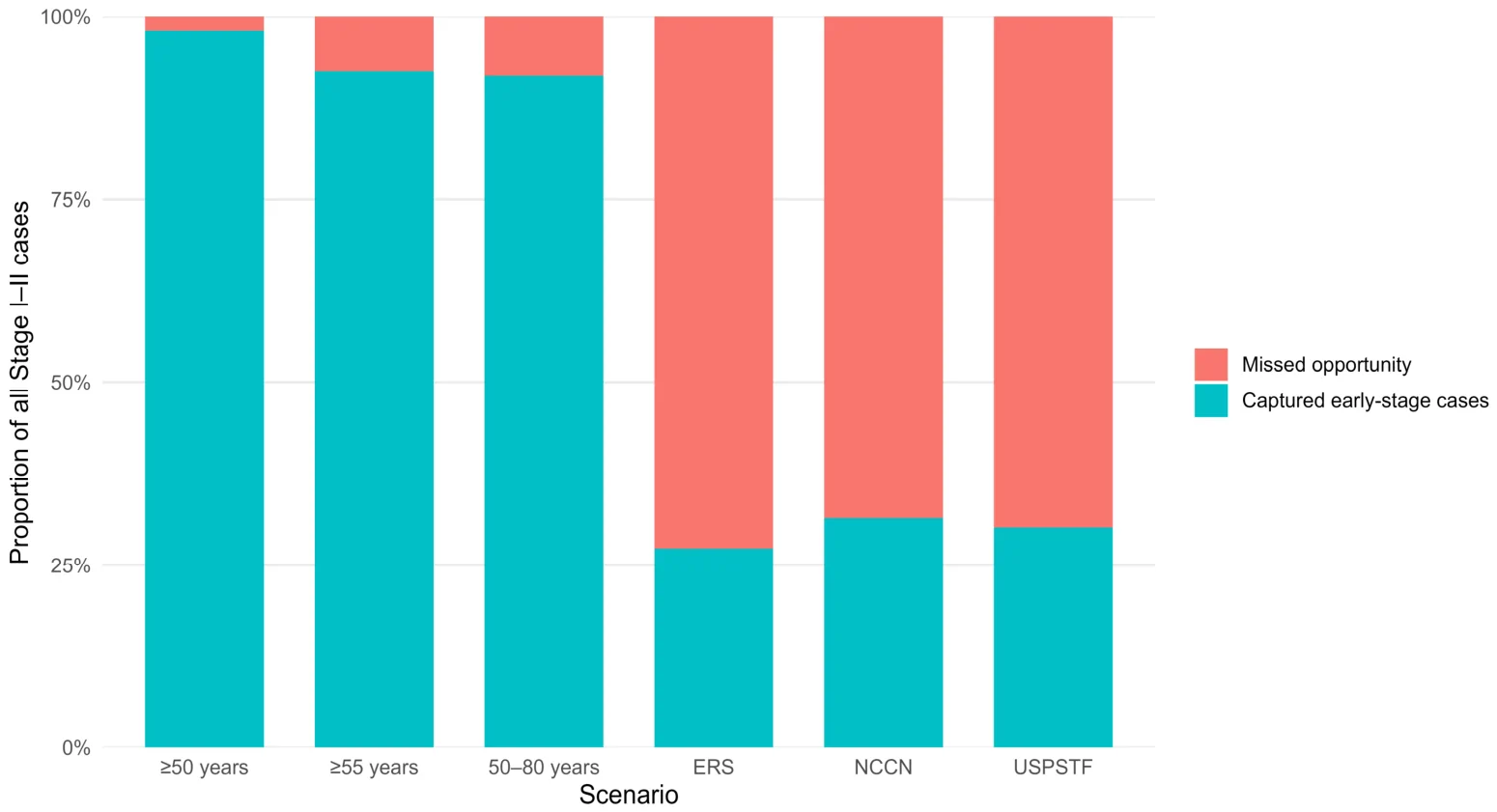
Captured early-stage cases versus missed opportunity across screening scenarios. Stacked bar chart illustrating the proportion of Stage I–II lung cancer cases captured by each eligibility scenario compared with the proportion missed (missed opportunity index, MOI). Age-only criteria demonstrate near-complete capture, whereas guideline-based models (USPSTF, NCCN, ERS) miss a substantial fraction of early-stage cases.

To further quantify the effectiveness of each screening scenario in identifying early-stage disease, we evaluated the proportion of Stage I–II cases captured versus those missed, expressed as the missed opportunity index (MOI) (Figure 5).

The comparative analysis of captured early-stage cases and missed opportunities reveals a marked divergence between theoretical age-only comparator scenarios and guideline-based screening strategies. Age-only scenarios demonstrate a near-complete capture of Stage I–II cases, with capture rates of 98.1%, 92.5%, and 91.8% for the ≥50 years, ≥55 years, and 50–80 years scenarios, respectively. The corresponding MOI values were 1.9%, 7.5%, and 8.2%.

In contrast, the incorporation of smoking-based eligibility criteria leads to a substantial reduction in retrospective early-stage case capture in this cohort. Specifically, guideline-based models exhibit high MOI values, ranging from 68.5% for NCCN to 72.7% for ERS, with USPSTF showing an MOI of 69.9%. Correspondingly, only 31.5%, 27.3%, and 30.1% of early-stage cases were retrospectively classified as eligible by NCCN, ERS, and USPSTF, respectively. This indicates that the majority of early-stage lung cancer cases in this cohort would have been classified as non-eligible under current screening eligibility frameworks. Notably, even the most inclusive guideline model (NCCN) has classified 98 of 143 early-stage cases (68.5%) as non-eligible.

These findings underscore a possible mismatch between existing screening eligibility paradigms and the risk profile of this tertiary-center cohort: while designed to target high-risk populations, their retrospective application to this cohort classified a large proportion of patients who ultimately present with potentially curable disease as non-eligible. The contrast with age-only criteria further highlights that restrictive exposure thresholds particularly those based on smoking history may significantly reduce retrospective eligibility among early-stage cases in this cohort.

These findings underscore a possible mismatch between existing screening eligibility paradigms and the risk profile of this tertiary-center cohort: while designed to target high-risk populations, their retrospective application to this cohort classified a large proportion of patients who ultimately present with potentially curable disease as non-eligible… suggesting the potential value of investigating broader or alternative risk stratification approaches, including multivariable prediction models, in prospective screening populations.

Sensitivity analyses demonstrated substantial quantitative dependence of screening eligibility and MOI estimates on the handling of missing smoking-related information (Supplementary Table S1). In the primary conservative analysis, MOI was 69.93%, 68.53%, and 72.73% for USPSTF, NCCN, and ERS, respectively. Excluding patients with indeterminate eligibility reduced these estimates to 30.65%, 13.46%, and 43.48%, while under best-case assumptions they decreased further to 13.29%, 4.90%, and 20.98%, respectively. Thus, although the magnitude of missed opportunity was strongly influenced by missing-data assumptions, missed early-stage cases persisted under all examined scenarios.

### 3.7. Hypothetical Screening Impact

**Figure 6:**
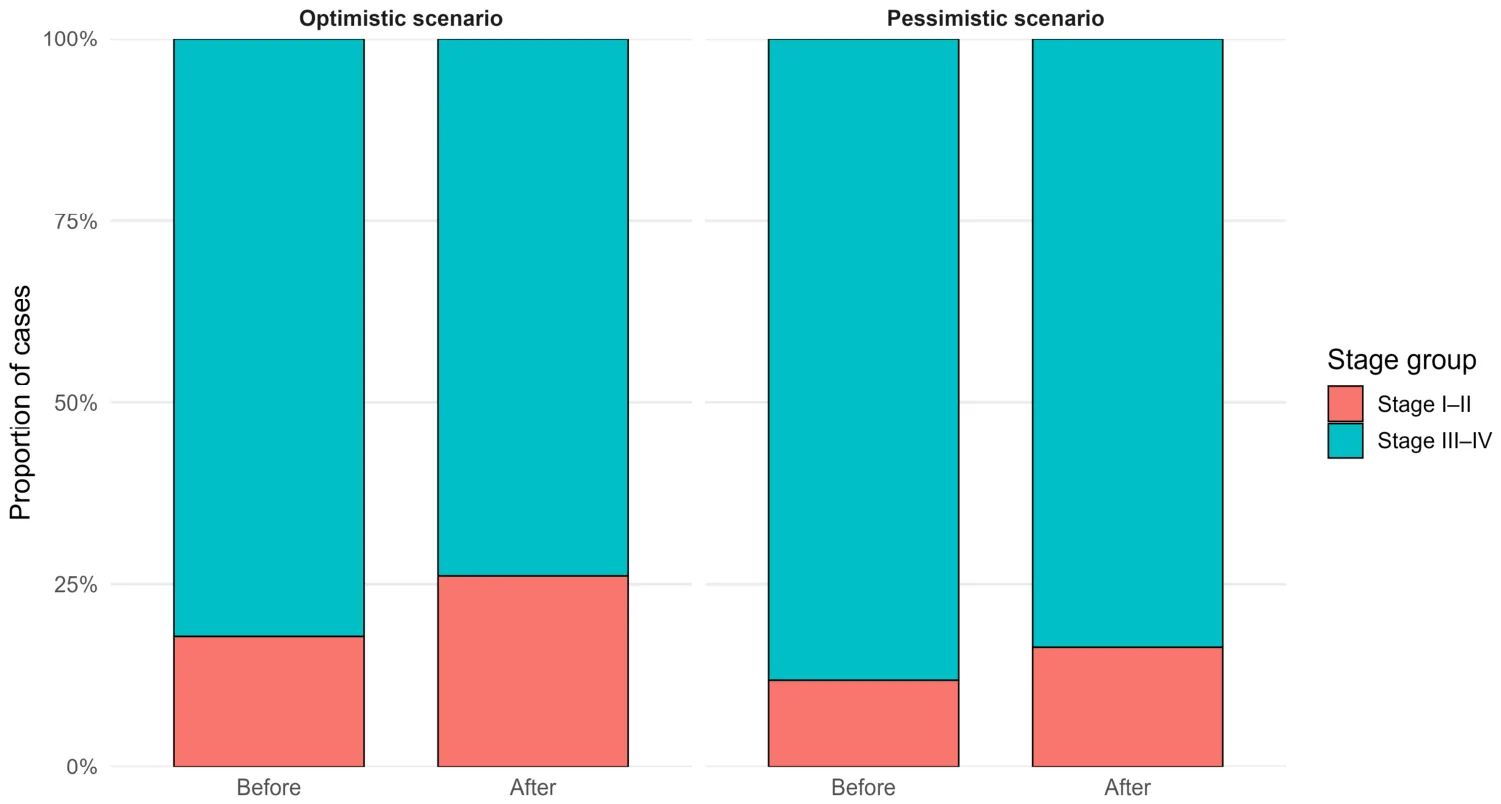
Hypothetical stage-shift under screening and sensitivity-analysis assumptions. Stacked bar plots illustrate stage-group distributions before and after a hypothetical screening-associated stage shift. In the optimistic scenario, missing-stage cases were assumed to follow the observed distribution among cases with available stage information, and 10% of Stage III–IV cases were subsequently reclassified as Stages I–II. In the pessimistic scenario, missing-stage cases were assigned as 10% Stages I–II and 90% Stages III–IV, followed by reclassification of 5% of Stage III–IV cases as Stage I–II cases. These percentages represent predefined scenario assumptions rather than effect estimates derived from the present cohort.

To explore the potential impact of screening under uncertainty related to missing stage data, a stage-shift simulation was performed, and the resulting changes in stage distribution are illustrated in Figure 6. In the optimistic scenario, the number of Stage I–II cases increased from 673 to 982, corresponding to an absolute gain of 309 cases (+8.2% of the total cohort). This was accompanied by a proportional reduction in Stage III–IV cases from 3091 to 2782. In the pessimistic scenario, a more conservative effect was observed, with Stage I–II cases increasing from 447 to 613 (+166 cases; +4.4%), and Stage III–IV cases decreasing from 3317 to 3151.

Despite differences in assumptions regarding missing data, both scenarios yielded qualitatively similar results, indicating that the modeled assumptions illustrate how even modest hypothetical stage shifts could translate into increases in the proportion of cases classified as Stages I–II. However, the magnitude of this benefit remains sensitive to the underlying distribution of unobserved cases.

## 4. Discussion

### 4.1. Current Landscape of Lung Cancer Screening and Management in Romania

#### 4.1.1. National Screening Initiative and Care Pathway

Although LDCT-based lung cancer screening is increasingly supported by European policy frameworks, its adoption across the EU remains inconsistent. Current evidence indicates that only a limited number of countries have implemented or are piloting organized screening programs, whereas the majority, Romania among them, have not yet established national screening pathways [31]. This disparity underscores ongoing challenges in translating trial evidence and policy recommendations into routine clinical practice, particularly in resource-constrained or structurally heterogeneous healthcare systems like Romania’s.

This heterogeneous implementation landscape is particularly relevant in the context of current European efforts to progressively integrate lung cancer screening into organized cancer prevention strategies. While several European countries have advanced toward national or regional LDCT screening programs, others remain at pilot, feasibility, or preparatory stages, resulting in substantial variation in population coverage, eligibility assessment, infrastructure, and integration with diagnostic and treatment pathways. Romania should therefore be considered within this broader European implementation continuum, with particular relevance to the challenges faced by Central and Eastern European healthcare systems in translating common policy objectives into locally feasible screening pathways.

In October 2024, the Romanian Ministry of Investments and European Projects, through the Managing Authority for the Health Programme, launched a public consultation on a national call for projects aimed at establishing and implementing a comprehensive lung cancer pathway. The initiative focuses on prevention, early detection through screening, diagnosis, and referral to treatment, within the framework of the EU-funded Health Programme. It aligns with broader European policy objectives to improve access to high-quality, preventive, and person-centered healthcare services, particularly at the primary and outpatient care levels. In July 2025, the same authority released the interim list of projects that passed the technical and financial evaluation stage for a national initiative aimed at lung cancer prevention, early detection through screening, and referral for diagnostic assessment [32]. This development marks a relevant administrative step toward the establishment of a structured national lung cancer screening framework in Romania and places the country within the broader, although still heterogeneous, transition toward organized LDCT-based screening across Europe.

Within this national initiative, a key component involves the structured training of personnel engaged in population identification, mobilization, and support for participation in lung cancer screening programs. This includes not only program coordinators and implementation staff, but also frontline healthcare and community personnel such as general practitioners, nurses, community health workers, health mediators, psychologists and social workers [32]. Strengthening this workforce is essential to ensure effective outreach, equitable access, and sustained patient engagement throughout the screening process.

Beyond initial screening participation, the continuity of care along the oncological pathway remains critical. In current practice in Romania, patients are typically referred to a specialist by general practitioners following suspicion of malignancy, after which diagnostic confirmation and treatment planning are coordinated by the oncologist. Optimal management relies on a multidisciplinary approach involving medical oncologists, thoracic surgeons, and radiation oncologists, ensuring that patients receive individualized, stage-appropriate care.

However, access to this pathway is influenced by structural disparities within the healthcare system. A significant proportion of the population resides in rural areas, where access to healthcare services is more limited. Healthcare infrastructure and workforce are disproportionately concentrated in urban settings, which host the vast majority of hospitals and specialist services, as well as a larger share of primary care practices [33]. This uneven distribution may have contributed to delays from symptom onset to specialist referral and diagnosis in our patient cohort, particularly in underserved regions.

Ultimately, while population-adapted screening should incorporate local environmental and host-specific factors, prioritizing individuals with high tobacco exposure remains paramount in high-prevalence settings. Improving population coverage through front-line community outreach is essential to ensure that screening effectively reaches these highest-risk segments.

#### 4.1.2. Imaging Infrastructure and Staging in Romania

Results from initial chest and upper abdomen CT and, where available, PET-CT were employed for accurate TNM staging, although in case of suspicion of metastatic disease, results of other imaging tests were assessed such as brain, abdominal or pelvic CT/MRI scans. While PET-CT is nowadays recommended for pre-treatment work-up, patients included in this study go back as far as 1990 when this investigation was not yet available. PET-CT first became available in Romania in 2008 in the private sector [34] and in 2010 it became reimbursable for oncological conditions through the national health insurance system [35]. As of January 2025, Romania has adopted the 9th edition of the TNM classification for lung cancer; however, as data for this study were collected in 2024, patient staging was performed according to the 8th edition. Given that the 8th TNM classification was introduced in 2017 [36], and to ensure consistency across cases diagnosed before its implementation, all patients from both cohorts with sufficient imaging and clinical data were retrospectively re-staged using the 8th edition.

#### 4.1.3. Access to Novel Cancer Therapies and Reimbursement

Another factor that may partly explain the absence of certain data from the included patient cohorts is the historical variability in access to EGFR, ALK, and PD-L1 testing. Although assessment of these biomarkers is now recommended as part of the diagnostic work-up for NSCLC in international guidelines, including those of the European Society for Medical Oncology [37], their availability in Romania has evolved gradually. Initial access was largely facilitated through private initiatives like industry-issued vouchers, beginning around 2016 [10], whereas reimbursement through the Romanian National Oncology Program has only become available more recently, since 2024, and encompasses testing panels for both SCC and non-SCC histologies [35]. Because both cohorts in this study include patients diagnosed with lung cancer as early as 1990, the lack of biomarker data is likely attributable, at least in part, to the limited availability of this testing during earlier diagnostic periods.

Access to innovative systemic therapies in Romania is limited by prolonged reimbursement timelines, although costs are covered by the national health insurance system. Once approved, most guideline-recommended treatments are available. For oncogene-driven NSCLC, osimertinib is reimbursed in both metastatic and adjuvant EGFR-mutant settings, including T790M-positive progression, while alectinib, brigatinib, and lorlatinib are available for ALK-positive disease, and crizotinib for ROS1 rearrangements [10].

In non-oncogene-driven NSCLC, immunotherapy is widely accessible, including pembrolizumab or atezolizumab monotherapy in PD-L1-high tumors, as well as chemo-immunotherapy or dual checkpoint blockade (nivolumab in combination with ipilimumab) regardless of PD-L1 status. Durvalumab is reimbursed as consolidation after chemoradiotherapy in unresectable stage III disease, and chemo-immunotherapy combinations are available in extensive-stage SCLC [10]. Overall, despite broad therapeutic availability, delays in reimbursement remain a key barrier to timely access.

### 4.2. The Diagnostic Gap and Late-Stage Presentation Reality

The high proportion of advanced-stage disease (Stages III and IV) observed in our cohort underscores a critical diagnostic gap that is highly representative of the broader CEE clinical landscape. In this region, the diagnostic pathway for lung cancer remains almost entirely symptom-driven rather than proactive, meaning that patients are typically identified only after the disease has progressed enough to cause significant clinical manifestations [8]. When correlating our cohort’s late-stage predominance with regional real-world data, the similarities are striking; for example, recent studies demonstrate that the vast majority of lung cancer patients in Romania and Bulgaria present with advanced disease, with over 60% of cases being diagnosed at Stage IV [8,11]. This overwhelming dominance of late-stage diagnoses highlights the fundamental failure of relying on symptomatic presentation in the absence of robust, population-level early detection programs.

Furthermore, the consequences of this symptom-driven approach are heavily exacerbated by profound diagnostic delays, which represent a systemic healthcare issue across the entire CEE region. Because early-stage lung cancer is largely asymptomatic, patient access to initial medical evaluation is inherently delayed, a problem frequently compounded by prolonged referral times, fragmented care, and bottlenecks between the initial diagnosis and the initiation of targeted treatment [38]. A comprehensive nationwide analysis from Hungary confirmed that the length of the patient pathway before first-line therapy is dangerously prolonged, with significant delays occurring at multiple steps of the diagnostic and therapeutic journey [38]. Ultimately, these systemic delays from symptom onset to first-line therapy inevitably push a massive proportion of patients into incurable stages, limiting therapeutic interventions to palliative care and driving the exceptionally poor survival outcomes observed in our cohort and throughout the wider region [38].

### 4.3. Structural Missingness and the Case for Centralized Registries

In our full analytical cohort (Set A) of 3764 patients, we observed a substantial missing data rate, with 71.4% lacking sufficient information for smoking-status classification and 82.8% lacking cumulative tobacco-exposure (pack-year) documentation. Specifically, key screening and therapeutic determinants such as smoking history were missing in more than 70% of cases, while molecular biomarkers like EGFR, ALK, and PD-L1 were unavailable in 76% to 80% of the records. Rather than viewing this high rate of missingness merely as a methodological limitation of our study, it must be interpreted as a prominent symptom of a profound systemic and structural deficit within the healthcare infrastructure. Since molecular testing was available only for a minority of patients and was not performed systematically, the observed frequencies and clinicodemographic associations may therefore reflect selective testing practices in addition to underlying biological patterns and should not be extrapolated to the full institutional cohort or the Romanian lung cancer population.

The root cause of this data fragmentation is the absence of a functional, comprehensive national lung cancer registry in Romania. Currently, cancer data in the country remains highly fragmented and non-standardized, forcing researchers, clinicians, and policymakers to rely on isolated institutional records [12]. This reliance inevitably leads to high missingness rates in real-world cohorts and severely limits the ability to accurately monitor cancer incidence, track patient pathways, formulate robust health policies, and support essential oncological research [10,12].

This infrastructural gap is particularly striking when contrasted with the centralized data ecosystems successfully implemented by neighboring CEE countries, which share similar historical and epidemiological backgrounds. For instance, Hungary utilizes its National Health Insurance Fund claim database, which covers nearly 100% of the population. This highly centralized system captures detailed information on in-patient and out-patient visits, allowing for robust, nationwide tracking of patient pathways, epidemiological shifts, and complex survival modeling [39,40]. Similarly, Poland has pioneered the National Cancer Network pilot program, which collects standardized clinical, pathological, and billing data in a central data warehouse. This allows for the precise calculation of quality measures for lung cancer diagnosis and enables continuous monitoring to improve treatment outcomes for advanced-stage patients [41,42]. These centralized systems generate the exact type of high-quality, population-level real-world evidence that Romania currently struggles to aggregate.

Despite these systemic challenges, the dual-cohort methodological approach utilized in this study proved robust. Our analysis demonstrated broad comparability between the full cohort (Set A) and the complete-case subset (Set B) across several demographic and exposure strata; for example, smoking-status and pack-year distributions across age and sex strata showed no significant between-cohort differences. However, complete and incomplete cases differed significantly in sex distribution (*p* < 0.001), while stage-group distribution did not differ significantly (*p* = 0.094). This overall pattern supports the use of Set B for analyses requiring more complete clinical information, while also indicating that complete-case selection was not entirely neutral across all baseline characteristics. Nevertheless, the sheer necessity of extracting a complete-case subset out of a massive pool of fragmented records exposes an urgent, unmet need for standardized clinical documentation. Consequently, establishing a comprehensive national lung cancer registry must be prioritized over immediate large-scale screening rollout. Without this centralized digital infrastructure, actual screening outcomes cannot be validated through longitudinal real-world data, and the potential downstream harms of screening—including overdiagnosis and false-positive procedures—cannot be adequately detected or monitored.

### 4.4. Geographic Disparities and Environmental Drivers

The pronounced urban predominance observed in our cohort, with 67.3% of patients originating from urban areas, raises a critical epidemiological question: does this skew reflect an actual higher oncological risk profile in cities, or is it primarily indicative of profound disparities in healthcare access? In transitioning CEE health systems, rural populations frequently experience systemic barriers such as geographic isolation from tertiary diagnostic centers, lower health literacy, and socioeconomic deprivation—that hinder timely diagnosis and referral. Recent spatial analyses of cancer incidence and mortality highlight these structural inequities, demonstrating that spatial clusters of high lung cancer mortality are strongly correlated with regional deprivation, and that rural populations may be significantly underrepresented in official incidence registries due to missed or post-mortem diagnoses [43].

One possible contextual explanation for the observed urban predominance is differential environmental exposure; however, individual exposure to air pollution, radon, or occupational carcinogens was not measured in the present cohort. Beyond traditional tobacco consumption, urban populations in this region are exposed to alarming levels of atmospheric pollution, primarily driven by coal combustion, vehicle emissions, and heavy industry. The severe impact of this pollution is well-documented in neighboring countries like Poland, where urban agglomerations experience exceptionally high concentrations of particulate matter (PM10, PM2.5) and carcinogenic polycyclic aromatic hydrocarbons (PAHs), such as benzo(a)pyrene. The magnitude of this exposure is so extreme that researchers estimate urban residents in Poland passively “smoke” the equivalent of roughly half a pack of cigarettes daily simply by inhaling ambient air, acting as a massive, non-traditional driver of lung cancer [44,45]. Consequently, environmental exposures represent a plausible contextual hypothesis for the observed geographic pattern, but the present data cannot determine their contribution relative to referral patterns, healthcare-access differences, or other unmeasured factors such as the synergistic carcinogenic burden of heavy smoking and chronic inhalation of urban PM10-bound heavy metals and PAHs.

Conversely, the reliance on data from tertiary urban centers risks the underrepresentation of unique rural-specific hazards. While urban areas face severe smog, specific rural micro-regions are heavily impacted by distinct environmental exposures that standard screening models fail to capture. A primary example is residential radon exposure, a potent radioactive carcinogen that significantly drives lung cancer incidence in specific geographic pockets, such as the Transylvania region in Romania and the Lublin region in eastern Poland [19,46]. Rural populations are also more frequently exposed to unregulated occupational hazards, such as agricultural chemicals and asbestos roofing, which multiply the baseline risk of tobacco smoking. Recognizing these contrasting geographic drivers is essential; an effective and scalable screening strategy must be flexible enough to account for the heavy PM2.5/PAH pollution in urban centers while integrating localized environmental risks, such as radon, for rural demographics.

### 4.5. The Missed Opportunity Index (MOI) and Contextual Considerations in Applying Western Screening Guidelines

The comparative analysis of the complete-case subset reveals a notable divergence between age-only screening scenarios and established guideline-based models. While age-only criteria captured nearly all stage I–II cases, the application of standard Western guidelines incorporating strict smoking history (such as USPSTF, NCCN, and ERS) generated high MOI values, indicating that a substantial proportion of early-stage lung cancer cases would have been retrospectively classified as non-eligible. However, these findings should be interpreted with caution, as the models were not applied in their intended prospective screening context.

One important explanatory factor is the inherent selection bias of the dataset, including referral, spectrum, survival, and documentation biases typical of retrospective, hospital-based cohorts. Standard screening criteria were developed and validated for asymptomatic, high-risk populations, whereas the present cohort reflects a real-world, predominantly symptomatic population diagnosed post hoc, often at more advanced stages. Applying eligibility thresholds designed for screening to a symptomatic population may therefore overestimate the proportion of early-stage cases retrospectively classified as non-eligible and does not represent true prospective screening performance. A critical reason why strict pack-year criteria may be less applicable in this population is potential exclusion of individuals who develop non-smoking-related lung cancers. Western screening models primarily capture smoking-associated tumorigenesis and may not fully reflect the evolving histological and molecular spectrum of the disease. Recent genomic profiling of Romanian NSCLC cohorts has uncovered a distinct molecular landscape characterized by a high prevalence of actionable driver mutations that fall outside the traditional screening demographics. Specifically, EGFR mutations have been detected in up to 14.3% of Romanian NSCLC patients, alterations that are overwhelmingly prevalent in non-smoking females and patients with adenocarcinoma [17,18]. While comprehensive genomic profiling is essential for guiding targeted therapies [4], reliance on smoking-based eligibility alone may not fully capture risk in non-smoking populations with a higher proportion of genetically driven disease.

In addition, the direct transfer of standard screening approaches into the Romanian healthcare setting may face an important contextual challenge: the “TB confounder” which accounts for the historically high and enduring incidence of TB in Romania [22]. On LDCT, these benign findings may resemble early-stage malignancies, potentially increasing false-positive rates if conventional size-based criteria are applied without contextual adaptation. Previous experience suggests that such diagnostic ambiguity can lead to unnecessary invasive procedures for benign disease [23]. Previous tuberculosis, granulomatous lesions, pulmonary nodule characteristics, and LDCT false-positive findings were not recorded at the patient level in the present cohort; therefore, this consideration is based on the regional epidemiological literature and should be regarded as an implementation hypothesis rather than an explanation directly demonstrated by our data.

Taken together, these findings do not indicate that Western screening models are ineffective, but rather that their applicability may be limited in this specific epidemiological and clinical context, suggesting a potential mismatch between standard eligibility models and the risk profile of this tertiary-center population. Prospective, population-based studies are needed to evaluate adapted or multivariable risk models in Romania and similar settings.

### 4.6. Stage-Shift Value and Optimizing Screening for CEE

The stage-shift simulation provides a hypothetical quantitative illustration of how earlier detection could alter the stage distribution of lung cancer under the predefined scenario assumptions. The modeled redistribution from advanced (Stage III–IV) to early-stage (Stage I–II) disease illustrates the potential magnitude of stage redistribution under the specified assumptions. Such a shift is not merely a statistical improvement but represents a fundamental transformation in clinical trajectories, as early-stage lung cancer is associated with significantly higher curability and survival rates, as well as reduced treatment complexity and cost.

Importantly, these findings must be interpreted in the context of the observed inefficiencies of current eligibility frameworks. While guideline-based selection may fail to capture a substantial proportion of cases, the present analysis suggests that, once implemented, screening itself remains a highly powerful intervention. In other words, even imperfect selection criteria may still yield a considerable population-level benefit if they succeed in advancing the timing of diagnosis. This reinforces the need for a pragmatic, regionally adapted screening model that prioritizes feasibility and cost-effectiveness, rather than strict adherence to externally derived criteria that may not fully reflect the risk structure of CEE populations.

A critical challenge in this region is the high prevalence of TB and other benign granulomatous diseases, which act as major confounders in LDCT-based screening and contribute to a high rate of indeterminate nodules and false positives. In this context, the integration of volumetric and dynamic criteria, particularly VDT, emerges as a key optimization strategy. Hungarian screening programs have demonstrated that VDT-based assessment is both highly cost-effective and clinically robust, enabling reliable differentiation between indolent or inactive TB-related lesions and progressively growing malignant nodules [5,6]. The adoption of such criteria could substantially reduce unnecessary follow-up procedures and improve the specificity of screening in TB-endemic settings.

In addition to imaging-based approaches, emerging evidence highlights the potential role of biological triage tools within the screening pathway. Biomarker-driven strategies, such as serum metabolomic profiling and circulating miRNA signatures, have shown potential in refining malignancy risk assessment for indeterminate nodules. Early screening initiatives, particularly from Poland, suggest that these approaches may help reduce unnecessary invasive procedures while preserving diagnostic performance [24,25]. The integration of these molecular tools into screening algorithms represents a natural evolution toward more precise and individualized risk assessment.

Finally, the full benefit of a stage shift can only be realized within a coordinated and patient-centered care pathway. Screening alone is insufficient if patients are subsequently lost to follow-up or experience delays in diagnostic resolution and treatment initiation. In fragmented healthcare systems, which are characteristic of many CEE countries, patient navigation programs play a pivotal role in ensuring continuity of care. Evidence indicates that structured navigation interventions significantly improve adherence to diagnostic and therapeutic pathways and are associated with improved survival outcomes [47]. Therefore, the implementation of screening programs must be accompanied by active patient navigation to translate early detection into tangible clinical benefit.

### 4.7. Limitations and Interpretation of Differences Between Model Types

The marked divergence observed between age-only and age + smoking-based screening models can be plausibly attributed to several intrinsic characteristics of the dataset. These factors reflect not only the epidemiological profile of the cohort, but also the manner in which real-world populations deviate from the assumptions embedded in guideline-derived risk models.

#### 4.7.1. High Proportion of Never-Smokers or Low-Exposure Individuals

A central explanation for the marked divergence observed between age-only models and established guideline-based screening criteria is the likely presence of a substantial subgroup of patients with either no smoking history or tobacco exposure falling well below standard guideline thresholds (e.g., <20 or <30 pack-years). Such individuals are systematically excluded by the restrictive criteria of major models such as the USPSTF, NCCN, and ERS, yet they may still develop lung cancer, including potentially curable early-stage disease.

This phenomenon is particularly relevant in Eastern European populations, where despite historically high smoking rates, the demographic and histological profiles of lung cancer are shifting. While cigarette smoking remains the primary driver of the disease, worldwide epidemiological data indicate that lung cancer in never-smokers is an emerging and distinct disease entity, accounting for approximately 15% to 25% of all cases globally [48,49,50]. In the Romanian context, recent real-world observations align with this trend, with some institutional cohorts reporting that up to 27% of patients diagnosed with lung cancer are non-smokers [22].

Furthermore, this diagnostic gap disproportionately affects female patients and is closely tied to an adenocarcinoma-predominant histological profile. Adenocarcinoma has become the leading cause of cancer-related deaths in never-smoking females and is frequently diagnosed in younger patients with light or no smoking history [51,52].

Thus, the clinical dataset includes a non-negligible burden of lung cancer that is biologically and epidemiologically unrelated to heavy smoking. Because current guideline-based models rely predominantly on smoking history as the gateway to early radiological detection, they systematically deny this genetically susceptible, non-smoking population the opportunity for a curative diagnosis. This contextual mismatch inevitably leads to the artificially high MOI values observed when applying smoking-centric frameworks like the USPSTF or NCCN criteria to real-world Eastern European cohorts.

#### 4.7.2. Shift in Histological Spectrum

The enrichment of adenocarcinoma cases within our dataset significantly contributes to the observed discrepancy between standard screening guidelines and the real-world capture of early-stage lung cancer. Globally, the histological spectrum of NSCLC has shifted over the past decades, with adenocarcinoma surpassing squamous cell carcinoma to become the most frequently diagnosed histological subtype in industrialized nations [53,54].

This histological shift heavily impacts the efficacy of traditional screening criteria because adenocarcinoma exhibits a distinct epidemiological profile. Most notably, adenocarcinoma is the most frequent histological type diagnosed in never-smokers and light smokers, particularly among female patients [51,53]. The pathogenesis in this demographic is frequently driven by specific molecular alterations, such as EGFR mutations and ALK rearrangements, which are overwhelmingly prevalent in non-smoking patients with adenocarcinoma rather than heavy smokers [17,55].

Furthermore, adenocarcinoma has a distinct anatomical presentation. Unlike squamous cell carcinomas, which typically arise centrally near the major bronchi and provoke earlier clinical symptoms (e.g., hemoptysis or airway obstruction), adenocarcinomas most often originate in the peripheral regions of the lung parenchyma [53,54]. Because of this peripheral location, they can remain clinically silent for longer periods but are highly susceptible to being detected incidentally at earlier stages when patients undergo chest imaging for unrelated medical or respiratory conditions [56].

Consequently, a substantial proportion of these early-stage, incidentally detected adenocarcinomas occur in individuals who lack a heavy tobacco exposure history. By definition, these patients fall entirely outside the classical high-risk definitions established by the USPSTF, NCCN, and ERS guidelines, which strictly gatekeep screening eligibility behind cumulative pack-year thresholds [57]. This contextual mismatch drastically reduces the capture rates of smoking-based models, driving up the MOI in modern cohorts and highlighting the urgent need for screening algorithms to adapt to the rising prevalence of peripherally located, non-smoking-related adenocarcinomas.

#### 4.7.3. Age Distribution Favoring Broad Eligibility

The application of age-only screening models (e.g., ≥50 years) to our retrospective cohort effectively included nearly the entire patient population, capturing approximately 95% of the analyzed cases. This broad eligibility strongly aligns with the well-established epidemiological consensus that lung cancer incidence is inherently correlated with advanced age. Across CEE, the vast majority of lung cancer cases are diagnosed in patients over 50, with incidence rates steadily increasing and peaking in the 60–79 age demographic [40,58,59].

While age-only criteria successfully identified almost all early-stage cases in our dataset, this phenomenon artificially maximizes early-stage capture. In a real-world oncology cohort where the median age is predominantly in the mid-sixties, utilizing such broad age threshold renders age a virtually non-discriminatory criterion [60]. Rather than providing precise risk stratification to differentiate truly high-risk individuals from the general population, the age-only model simply reflects the underlying disease prevalence and the natural biological accumulation of cancer risk over time [61,62].

Thus, the apparently superior performance of age-only scenarios in capturing Stage I-II disease is largely structural. It arises from the near-universal inclusion of the symptomatic cohort rather than indicating true predictive efficiency or diagnostic specificity [57]. This highlights a significant limitation in evaluating screening models retrospectively: casting an exceptionally wide net inevitably captures more cases, but in a prospective, asymptomatic population, such a non-specific approach would result in an unsustainable volume of screening scans and false-positive investigations [63]. Accordingly, these age-only scenarios should be regarded as theoretical comparators rather than realistic screening proposals; their clinical utility cannot be inferred from the present analysis and would require formal prospective evaluation of specificity, false-positive findings, downstream diagnostic procedures, potential anxiety, resource utilization, and cost-effectiveness.

#### 4.7.4. Late Clinical Presentation Despite Eligibility

A critical observation from our analysis is that even among individuals who successfully meet the stringent age and smoking-based criteria of established screening guidelines (e.g., USPSTF, NCCN, and ERS), a remarkably large proportion still present with advanced-stage disease. In our dataset, advanced-stage cases (Stages III–IV) comprised approximately 85% to 86% of the theoretically eligible patients, with only a modest fraction diagnosed at an early, curable stage (Stages I–II).

This persistent predominance of late-stage disease among “high-risk” eligible individuals strongly suggests that the dataset reflects a clinically diagnosed, symptomatic population rather than an actively screened, asymptomatic one. In the real-world CEE setting, early-stage lung cancer is frequently clinically silent, meaning that most patients seek medical care only after the onset of severe respiratory or systemic symptoms, by which time the disease has already progressed to an incurable stage [64].

The failure to intercept the disease earlier in these eligible patients points to profound systemic barriers. Across the region, there are well-documented delays in the diagnostic pathway, characterized by prolonged intervals between symptom onset, primary care referral, specialized diagnostic workup, and the initiation of first-line therapy [13,38]. Moreover, this advanced presentation heavily reflects the limited access to early detection infrastructure. Countries like Romania and Bulgaria currently lack organized, nationwide LDCT screening programs, relying instead on opportunistic and fragmented care [8,11]. Even in global or regional instances where screening pilots exist, the uptake remains notoriously low due to financial constraints, fear and stigma associated with a cancer diagnosis, low health literacy, and limited geographic access [65,66].

As a result, theoretical screening eligibility does not spontaneously translate into early detection in practice. Without a proactive, well-coordinated screening framework and effective patient navigation, even the most accurately selected high-risk individuals will continue to be diagnosed too late. This reality fundamentally weakens the apparent benefit of guideline-based selection criteria in retrospective cohorts, underscoring that defining the right target population must be coupled with overcoming the systemic barriers to screening access to achieve a meaningful stage shift.

#### 4.7.5. Incomplete or Inaccurate Smoking Data

A major methodological challenge in evaluating screening guidelines within our study—and a prominent feature of retrospective, hospital-based cohorts in general—is the profound limitation surrounding the quality of lifestyle documentation. Real-world datasets frequently suffer from a high rate of missing smoking history, as institutional medical records are typically optimized for acute treatment pathways rather than long-term epidemiological tracking [67,68].

Furthermore, even when a patient’s smoking status is documented as “current” or “former,” there is often an imprecise quantification of cumulative pack-years and a lack of detail regarding smoking duration or intensity [15]. Equally problematic is the frequent absence of unrecorded cessation timing (the exact number of “quit years”), which is a critical variable in modern screening algorithms [69]. Obtaining a precise, standardized smoking history is not readily available in most non-trial settings, a systemic issue compounded by the fact that many countries lack population-wide databases capturing such metrics [5,65].

These limitations severely distort the retrospective application of screening criteria. Because established models like the USPSTF, NCCN, and ERS depend heavily on strict pack-year thresholds and precise cessation windows, missing or underestimated smoking data inevitably lead to the misclassification of otherwise eligible individuals as non-eligible [57].

Consequently, this data deficit directly contributes to an artificial inflation of the MOI. While risk prediction models have been shown to improve selection accuracy over simple age and pack-year criteria [70], they still require robust baseline data. In retrospective hospital-based cohorts, unless smoking histories are prospectively gathered or undergo intensive manual curation [14], evaluating the true efficacy of smoking-based screening criteria will remain fundamentally impaired by the incomplete nature of real-world clinical records.

#### 4.7.6. Absence of Additional Risk Determinants

Established guideline-based lung cancer screening models, such as those endorsed by the USPSTF, NCCN, and ERS, rely predominantly on just two variables to define eligibility: chronological age and cumulative smoking exposure (pack-years) [57,65]. While smoking is undeniably the primary driver of the disease, this simplified approach fails to account for the complex, multifactorial etiology of malignancy. Our real-world dataset likely includes a significant number of patients whose tumorigenesis was heavily influenced by alternative, non-tobacco risk determinants that are highly prevalent in the region. Unfortunately, several potentially relevant risk determinants discussed in this manuscript, including individual radon, occupational, and air-pollution exposures, were not available or were very scarce at the patient level. Their potential contribution to the observed patterns therefore cannot be evaluated in the present study, and discussion of these factors is based on external epidemiological evidence rather than direct cohort-level associations.

First, CEE populations face substantial occupational and residential exposures to known carcinogens. Historical and ongoing occupational exposure to asbestos, particularly in rural and industrial settings, remains a potent trigger for respiratory system cancers [71,72]. Similarly, residential exposure to high indoor concentrations of radioactive radon gas is a major, often overlooked etiology, acting as the leading cause of lung cancer in non-smokers and demonstrating a synergistic carcinogenic effect with tobacco smoke [46,73].

Second, environmental pollution constitutes a massive, population-wide risk factor. Exposure to severe outdoor air pollution—specifically fine particulate matter (PM2.5, PM10), nitrogen dioxide, and PAHs—independently drives lung cancer incidence, particularly in heavily industrialized and urbanized agglomerations in countries like Poland and Romania [44,45,74].

Third, host-specific intrinsic factors, such as genetic susceptibility and pre-existing chronic lung diseases, heavily alter an individual’s cancer risk independently of their smoking status. A strong family history of lung cancer among first-degree relatives is a proven, powerful predictor of the disease, suggesting underlying genetic or shared environmental susceptibilities that standard guidelines ignore [75,76]. Furthermore, patients burdened with chronic lung diseases, most notably COPD and pulmonary fibrosis, face a markedly multiplied risk of lung cancer due to chronic inflammation, premature lung aging, and shared epigenetic alterations [21,53].

Because these critical risk determinants (occupational, environmental, genetic, and comorbid) are not captured by standard criteria, relying solely on age and smoking history leads to a systematic under-selection of highly vulnerable, at-risk individuals. This contextual mismatch inevitably drives up the MOI in real-world cohorts, highlighting the urgent need to adopt comprehensive, multivariable risk prediction models that integrate these alternative epidemiological factors to optimize screening eligibility [70].

#### 4.7.7. Selection Bias of a Symptomatic Cohort

A fundamental limitation to consider when evaluating the performance of established screening models in our study is the profound selection bias inherent to the dataset. The analyzed cohort represents a real-world clinical diagnostic population, composed almost entirely of symptomatic patients who sought medical care due to the onset of respiratory or systemic manifestations, rather than an actively monitored, asymptomatic screening population [64]. Accordingly, the high MOI observed in the primary analysis should not be interpreted as a precise estimate of guideline performance, because its magnitude is strongly influenced by missing smoking and pack-year information. Sensitivity analyses nevertheless demonstrated residual missed opportunities even under favorable assumptions, supporting the qualitative conclusion that smoking-based eligibility criteria alone would not capture all early-stage cases in this cohort.

This distinction introduces a critical structural bias into our retrospective analysis. Major lung cancer screening models, such as those endorsed by the USPSTF and NCCN, were rigorously designed and statistically validated to identify early, pre-clinical malignancies specifically in asymptomatic, high-risk individuals. These guidelines are grounded in data from highly controlled, proactive screening cohorts, such as NLST and the NELSON trial [77,78]. In stark contrast, our dataset reflects a clinical reality of *post hoc* diagnoses, where patients overwhelmingly present at advanced, incurable stages (Stage III and IV) driven by the late manifestation of the disease [8]. In real-world observational studies, relying on symptomatic presentation is consistently associated with severe systemic diagnostic delays and “wait-time” paradoxes, further differentiating this diagnostic cohort from an actively screened one [38,47].

Therefore, the apparent inefficiency of these guideline-based screening criteria, and the resulting MOI observed in our study, cannot be solely attributed to the biological or epidemiological shortcomings of the models themselves. Instead, it is partly the result of a profound contextual mismatch between the preventive, early-detection intent of the screening models and the late-stage, symptom-driven structure of the analyzed dataset. Evaluating screening efficacy by retrospectively applying predictive criteria designed for asymptomatic individuals to a symptomatic, advanced-stage cohort will inevitably underestimate the models’ potential preventative utility while artificially amplifying their failure rates.

#### 4.7.8. Demographic and Regional Particularities

Local epidemiological features and population dynamics significantly contribute to the discrepancies observed between our real-world dataset and the theoretical performance of established lung cancer screening models. The CEE, including Romania, exhibits a distinct epidemiological landscape that deviates considerably from the Western European and North American populations upon which major screening guidelines were validated.

A primary factor is the profound differences in smoking patterns—specifically the balance between smoking intensity and duration. Historically, the tobacco epidemic in CEE countries followed a different temporal and cultural trajectory. For instance, the widespread adoption of smoking among women occurred much later than in Western countries, leading to a delayed but sharp ongoing rise in female lung cancer incidence that traditional pack-year models may fail to accurately capture [79,80]. Additionally, regional clustering analyses have identified unique phenotypes, such as “extreme heavy smokers” heavily concentrated in rural areas, whose smoking intensity and synergistic respiratory comorbidities drastically outpace standard high-risk definitions [14].

Furthermore, this region faces a higher prevalence of non-smoking-related lung cancer driven by local genetic and environmental drivers. As noted previously, Romanian NSCLC cohorts demonstrate a high frequency of EGFR mutations (often found in non-smoking females), indicating a strong genetic susceptibility pathway that falls entirely outside tobacco-centric screening criteria [17,18]. This is exacerbated by severe regional environmental hazards. Urban populations in CEE countries are exposed to some of the highest levels of particulate matter (PM2.5, PM10) and carcinogenic PAHs in Europe, effectively rendering inhabitants passive “smokers” by environmental default [44,81]. Concurrently, rural populations in specific geographies, such as Transylvania, face significant, often unmeasured exposure to residential radon, a potent carcinogen acting independently of tobacco [46,82].

Finally, there is immense variability in healthcare access and diagnostic pathways. Unlike the populations enrolled in the NLST or NELSON trials, patients in transitioning CEE healthcare systems frequently encounter fragmented care, a lack of structured preventive medicine, and severe shortages of specialized oncological infrastructure in non-metropolitan areas [13,65].These systemic barriers lead to profound diagnostic delays—from the onset of symptoms to specialist referral and the initiation of treatment—which disproportionately affect rural and socioeconomically disadvantaged groups [8,38,83]. Consequently, the patient journey in this region is fundamentally skewed toward late-stage, symptomatic presentation rather than early, incidental detection.

Collectively, these factors may render international guidelines (such as those from the USPSTF, NCCN, and ERS) significantly less transferable to the specific population represented in our dataset [57]. The direct application of Western, smoking-centric models to a population burdened by different tobacco habits, severe environmental pollution, distinct genetic profiles, and delayed healthcare pathways inevitably leads to the under-selection of truly at-risk individuals. Therefore, to achieve a meaningful reduction in mortality, screening policies in Romania must pivot toward population-adapted, multivariable risk models that reflect these unique demographic and regional particularities.

In this context, the landmark lung cancer screening experiences from Asian cohorts—most notably in Taiwan, where screening has shifted from mass screening to specific risk-based models targeting never-smoking females with a family history of lung cancer—offer invaluable clinical and methodological parallels. These cohorts demonstrate that while expanding screening eligibility to non-smokers successfully captures early-stage disease in genetically susceptible groups, it simultaneously introduces substantial challenges regarding potential overdiagnosis and overmanagement, particularly among younger females. Mitigating these harms requires high-threshold risk-prediction models and conservative nodule management protocols. Within the framework of the European SOLACE project, which is dedicated to developing customizable, regional screening toolboxes that balance clinical benefits against potential overmanagement risks, these international insights are highly relevant. Synthesizing the overdiagnosis lessons from East Asia with CEE-specific environmental (radon, particulate matter) and infectious (tuberculosis) confounders allows the SOLACE project to design robust, multi-tiered risk-stratification pathways that protect vulnerable populations from under-selection while safeguarding the healthcare infrastructure from false-positive over-testing [84,85,86].

## 5. Conclusions

The retrospective analysis of this real-world Romanian tertiary cohort highlights a substantial diagnostic burden, with most lung cancer cases presenting at advanced stages. When exploring theoretical screening eligibility within this population, differences between age-based and smoking-based criteria were observed. However, these findings should be interpreted with caution, as they are derived from a retrospective, predominantly symptomatic cohort and do not reflect prospective screening performance.

Importantly, these results do not indicate that guideline-based screening approaches are ineffective. Rather, they suggest a potential mismatch between simplified eligibility criteria and the complex epidemiological profile observed in this setting. Factors such as the presence of non-smoking-related cancers, environmental exposures, and region-specific clinical confounders may not be fully captured by standard models, although these observations require validation in appropriate screening populations.

In addition, the analysis was affected by incomplete clinical and diagnostic data, which may have influenced the estimation of eligibility and stage distribution. This further underscores the need for cautious interpretation of the findings.

Overall, the study highlights the need for prospective, population-based research to evaluate the performance of screening strategies in Romania and similar Central and Eastern European settings. Such studies should aim to assess whether adapted or multivariable risk models can improve the identification of high-risk individuals while maintaining an appropriate balance between benefits and harms.

## Figures and Tables

**Table 1:** Overview of variables collected and analyzed in the study.

Domain	Variables Included
A. Demographic variables	Age; Sex;
B. Clinical and tumor variables	Histological subtype; tumor, node, metastasis (TNM) components (T, N, M) *; Derived stage (pathological tumor, node, metastasis (pTNM)_staging); Metastatic sites
C. Molecular variables	EGFR mutation status; anaplastic lymphoma kinase (ALK) rearrangement; programmed death-ligand 1 (PD-L1) expression
D. Risk factor variables	Smoking status; Pack-years; Occupational exposures; Environmental exposures
E. Treatment variables	Surgery; Radiotherapy (RT); Chemotherapy (CHT); Immunotherapy
F. Health-system/pathway variables (proxies)	Diagnosis Year; Treatment initiation (proxy for downstream diagnostic steps)
G. Geographic variables	Living conditions (urban/rural), county

* TNM 8th edition stage classification was used.

**Table 2:** Comparison of lung cancer screening eligibility criteria according to NCCN, ERS, and USPSTF guidelines.

	NCCN *	ERS **	USPSTF ***
Age criteria	≥50 years (category 1)	50–75 years	50–80 years
Smoking history and/or Pack-years	≥20 years of smoking cigarettes (category 2B) Or ≥20 pack-year smoking history (category 1)	Current or former smoker And ≥20 pack-year smoking history	20 pack-year smoking history And Current smoker Or Former smoker (quit within the past 15 years)

* NCCN Clinical Practice Guidelines in Oncology (NCCN Guidelines^®^) for Lung Cancer Screening V.1.2026. © National Comprehensive Cancer Network, Inc. 2026. All rights reserved. Accessed [10 May 2026]. ** Revel, M.-P.; Biederer, J.; Nair, A.; Silva, M.; Jacobs, C.; Snoeckx, A.; Prokop, M.; Prosch, H.; Parkar, A.P.; Frauenfelder, T.; et al. ESR Essentials: Lung Cancer Screening with Low-Dose CT—Practice Recommendations by the European Society of Thoracic Imaging. Eur Radiol 2025, 36, 2064–2073, doi:10.1007/s00330-025-11910-9. *** US Preventive Services Task Force Screening for Lung Cancer: US Preventive Services Task Force Recommendation Statement. JAMA 2021, 325, 962–970, doi:10.1001/jama.2021.1117.

**Table 3:** Data availability and proportion of missing values across key demographic, clinical, and molecular variables in the full cohort (Set A, n = 3764).

Variable	Available n	Missing n	Missing (%)
Age	3764	0	0
Sex	3764	0	0
Stage	889	2875	76.38
Histology	2161	1603	42.59
Smoking	1118	2646	70.30
EGFR	888	2876	76.41
ALK	779	2985	79.30
PD-L1	751	3013	80.05

EGFR—epidermal growth-factor receptor; ALK—anaplastic lymphoma kinase; PD-L1—programmed death ligand 1.

**Table 4:** Comparison of baseline demographic and tumor characteristics between complete cases (Set B) and patients with incomplete data (Set A), with separate reporting of “other” and missing histological categories.

Variable	Set B	Set A	*p*-Value
Age (years)	66.3 ± 8.9	66.9 ± 9.4	<0.001
Sex			0.092
Male	1253 (70.9%)	2617 (69.5%)	
Female	515 (29.1%)	1147 (30.5%)	
Stage group			0.728
Stages I–II	143 (8.1%)	159 (4.2%)	
Stages III–IV	663 (37.5%)	730 (19.4%)	
Missing	962 (54.4%)	2875 (76.4%)	
Histology			<0.001
Adenocarcinoma	944 (53.4%)	1130 (30.0%)	
SCC	453 (25.6%)	541 (14.4%)	
SCLC	199 (11.3%)	238 (6.3%)	
Other specified histologies	172 (9.7%)	252 (6.7%)	
Missing/unspecified	0 (0.0%)	1603 (42.6%)	

Values are mean ± SD for age and n (%) for categorical variables. Displayed percentages use the full Set B and full Set A cohort sizes as denominators. Because Set B is nested within Set A, inferential *p*-values do not compare Set A directly with Set B. *p*-values compare Set B with the independent Set A incomplete partition (Set A minus Set B). Age was compared using Welch’s *t*-test; categorical variables were compared using Pearson’s chi-square test or Fisher’s exact test when required by small expected counts. SCC—squamous cell carcinoma; SCLC—small-cell lung cancer.

**Table 5:** Baseline demographic and risk factor characteristics in the full cohort (Set A) and the complete-case subset (Set B).

Variable	Set A (n, %)	Set B (n, %)
Age categories (years)		
<50	174 (4.6%)	75 (4.2%)
50–59	559 (14.9%)	277 (15.7%)
60–69	1483 (39.4%)	749 (42.4%)
≥70	1548 (41.1%)	667 (37.7%)
Sex		
Male	2617 (69.5%)	1253 (70.9%)
Female	1147 (30.5%)	515 (29.1%)
Smoking status		
Current smoker	728 (19.3%)	602 (34.0%)
Former smoker	255 (6.8%)	206 (11.7%)
Never smoker	92 (2.4%)	72 (4.1%)
Missing	2689 (71.4%)	888 (50.2%)
Pack-years		
≥30 pack-years	485 (12.9%)	391 (22.1%)
<30 pack-years	162 (4.3%)	138 (7.8%)
Missing	3117 (82.8%)	1239 (70.1%)

**Table 6:** Comparative distribution of smoking status and cumulative tobacco exposure (pack-years) across age categories and sex between the full cohort (Set A) and the complete-case subset (Set B).

Category	Set A		Set B		Set A vs. B
	n (%)	*p*-Value	n (%)	*p*-Value	*p*-Value
Smoking status					
Age categories					0.998
<50 years		0.111		0.123	
Current smoker	33 (86.8%)		15 (83.3%)		1.000
Former smoker	5 (13.2%)		3 (16.7%)		
50–59 years					
Current smoker	116 (78.9%)		102 (79.1%)		0.974
Former smoker	31 (21.1%)		27 (20.9%)		
60–69 years					
Current smoker	322 (72.9%)		268 (72.8%)		0.994
Former smoker	120 (27.1%)		100 (27.2%)		
≥70 years					
Current smoker	257 (72.2%)		206 (73.0%)		0.809
Former smoker	99 (27.8%)		76 (27.0%)		
Sex					0.607
Male		0.269		0.947	
Current smoker	567 (73.3%)		474 (74.3%)		0.659
Former smoker	207 (26.7%)		164 (25.7%)		
Female					
Current smoker	161 (77.0%)		128 (75.3%)		0.692
Former smoker	48 (23.0%)		42 (24.7%)		
Pack-years					
Age categories					0.996
<50 years		<0.001		<0.001	
≥30 pack-years	11 (42.3%)		8 (40.0%)		0.875
<30 pack-years	15 (57.7%)		12 (60.0%)		
50–59 years					
≥30 pack-years	63 (63.6%)		55 (62.5%)		0.872
<30 pack-years	36 (36.4%)		33 (37.5%)		
60–69 years					
≥30 pack-years	220 (80.6%)		181 (81.2%)		0.870
<30 pack-years	53 (19.4%)		42 (18.8%)		
≥70 years					
≥30 pack-years	191 (76.7%)		147 (74.2%)		0.547
<30 pack-years	58 (23.3%)		51 (25.8%)		
Sex					0.973
Male		<0.001		<0.001	
≥30 pack-years	411 (80.7%)		330 (79.5%)		0.641
<30 pack-years	98 (19.3%)		85 (20.5%)		
Female					
≥30 pack-years	74 (53.6%)		61 (53.5%)		0.986
<30 pack-years	64 (46.4%)		53 (46.5%)		

Values are n (%) within each stratum and cohort. Smoking-status comparisons include current and former smokers only; never smokers and cases with missing smoking status are excluded from these comparative distributions. Pack-year comparisons include only cases with known cumulative tobacco exposure and use the <30 versus ≥30 pack-year classification. *p*-values correspond to row-wise Set A versus Set B comparisons within each stratum using Pearson’s χ^2^ test or Fisher’s exact test when required by small expected counts. Column-wise *p*-values within-cohort tests assess smoking-status or pack-year distribution across age categories or sex.

**Table 7:** Distribution of histological subtypes across demographic, clinical, exposure, and geographic variables in the complete-case subset (Set B).

Variable	Category	Adenocarcinoma (%)	SCC (%)	SCLC (%)	Others (%)	*p*-Value (Overall)
Sex	Female	66.8	14.2	9.9	9.1	<0.001
	Male	47.9	30.3	11.8	10.0	
	*p*-value	<0.001	<0.001	0.249	0.584	
Age categories	<50	73.3	10.7	9.3	6.7	0.003
	50–59	54.2	22.0	11.9	11.9	
	60–69	51.5	25.5	13.2	9.7	
	≥70	52.9	28.9	9.0	9.1	
	*p*-value	0.004	0.002	0.082	0.464	
Smoking status	Current	46.7	31.1	12.8	9.5	0.001
	Former	49.0	28.6	11.2	11.2	
	Never	73.6	16.7	1.4	8.3	
	*p*-value	<0.001	0.039	0.016	0.710	
Pack-years	<30	55.1	20.3	14.5	10.1	0.007
	≥30	40.9	34.3	12.0	12.8	
	*p*-value	0.004	0.002	0.453	0.413	
Staging categories	Stages I–II	54.5	29.4	3.5	12.6	0.004
	Stages III–IV	45.1	32.6	13.0	9.4	
	*p*-value	0.040	0.456	0.001	0.240	
Living environment	Urban	54.2	24.6	11.5	9.8	0.560
	Rural	51.9	27.7	10.8	9.6	
	*p*-value	0.358	0.155	0.675	0.920	

Values represent row-wise percentages among cases with available histology and the corresponding stratification variable. Overall *p*-values compare the full four-category histological distribution across levels of each variable. Supplementary *p*-values in the category-specific *p*-value rows compare each histological subtype against all remaining histological subtypes across the corresponding strata. Pearson’s chi-square test was used when expected counts were adequate; Fisher’s exact test or Monte Carlo Fisher testing was used when expected counts were small. SCC—squamous cell carcinoma; SCLC—small-cell lung cancer.

**Table 8:** Distribution of TNM stages (Stages I–IV) across demographic, exposure, and geographic variables in the complete-case subset (Set B).

Variable	Category	Stage I (%)	Stage II (%)	Stage III (%)	Stage IV (%)	*p*-Value (Overall)
Sex	Female	15.6	7.6	31.7	45.1	<0.001
	Male	6.4	9.3	40.2	44.2	
	*p*-value	<0.001	0.448	0.026	0.812	
Age categories	<50	3.1	3.1	53.1	40.6	0.316
	50–59	7.5	8.3	33.1	51.1	
	60–69	9.1	7.4	40.1	43.5	
	≥70	10.0	11.4	35.6	42.9	
	*p*-value	0.634	0.242	0.122	0.390	
Smoking status	Current	8.4	9.1	39.6	42.9	0.875
	Former	9.4	11.3	39.6	39.6	
	*p*-value	0.754	0.503	0.998	0.561	
Pack-years	<30	12.3	8.2	37.0	42.5	0.946
	≥30	10.2	7.3	39.3	43.2	
	*p*-value	0.613	0.794	0.725	0.913	
Living environment	Urban	10.0	8.7	37.3	44.1	0.548
	Rural	6.9	9.1	38.9	45.1	
	*p*-value	0.147	0.839	0.653	0.782	

Values represent row-wise percentages among patients with available TNM stage and the corresponding stratification variable. Overall *p*-values correspond to χ^2^ tests across all stage categories, while supplementary *p*-values indicate stage-specific comparisons. Supplementary *p*-values in the stage-specific *p*-value rows compare each individual stage against all remaining stages. The smoking-status analysis includes current and former smokers only; never smokers and cases with missing smoking status are excluded from that specific comparison. Pack-year analyses include only patients with known cumulative tobacco exposure. Pearson’s chi-square test was used when expected counts were adequate; Fisher’s exact test or Monte Carlo Fisher testing was used when expected counts were small.

**Table 9:** Distribution of molecular alterations (EGFR, ALK, PD-L1) across demographic, exposure, staging, and geographic variables in the complete-case subset (Set B).

Variable	Category	EGFR+ (%)	ALK+ (%)	PD-L1 ≥ 50% (%)	*p*-Value (Overall)
Sex	Female	34.0	12.4	19.6	<0.001
	Male	16.6	4.7	22.8	
	*p*-value	<0.001	<0.001	0.352	
Age categories	<50	14.0	25.6	31.6	0.003
	50–59	17.6	8.0	22.7	
	60–69	21.5	5.9	21.3	
	≥70	27.0	5.4	20.5	
	*p*-value	0.070	0.002	0.482	
Smoking status	Never/Former	17.8	7.1	26.5	0.311
	Current	14.2	6.3	19.4	
	*p*-value	0.344	0.745	0.111	
Pack-years	<30	25.4	3.7	21.8	0.056
	≥30	9.8	7.8	21.1	
	*p*-value	0.005	0.514	0.907	
Staging categories	Stages I–II	18.0	5.1	16.7	0.853
	Stages III–IV	19.9	6.0	23.5	
	*p*-value	0.752	1.000	0.356	
Living environment	Urban	24.8	7.8	21.9	0.156
	Rural	17.6	6.1	21.6	
	*p*-value	0.024	0.419	0.920	

Percentages are calculated among patients tested for each biomarker. Overall *p*-values correspond to χ^2^ tests across all categories, while per-marker *p*-values indicate biomarker-specific comparisons. The *p*-value row contains biomarker-specific association tests. Because EGFR, ALK, and PD-L1 have different tested-patient denominators, the displayed overall *p*-value is an omnibus Fisher combined-probability *p*-value derived from the three biomarker-specific association tests rather than a single ordinary contingency-table chi-square. Because molecular testing was not performed systematically, these biomarker-specific frequencies describe the tested subsets and should not be interpreted as prevalence estimates for the full cohort. EGFR—epidermal growth-factor receptor; ALK—anaplastic lymphoma kinase; PD-L1—programmed death ligand 1.

**Table 10:** Screening eligibility under three age-based minimal models (≥50 years, ≥55 years, and 50–80 years) in the full cohort (Set A, 3764 subjects).

Scenario	Category	n	%
≥50 years	Eligible	3590	95.38
	Not eligible	174	4.62
Subjects for which staging was available	Stages I–II among eligible	156	18.29
	Stages III–IV among eligible	697	81.71
≥55 years	Eligible	3379	89.77
	Not eligible	385	10.23
Subjects for which staging was available	Stages I–II among eligible	147	18.49
	Stages III–IV among eligible	648	81.51
50–80 years	Eligible	3352	89.05
	Not eligible	412	10.95
Subjects for which staging was available	Stages I–II among eligible	146	17.87
	Stages III–IV among eligible	671	82.13

**Table 11:** Screening eligibility proportions and stage distribution among eligible patients (Set B, 1768 subjects) according to three guideline-based models (USPSTF, NCCN, ERS). Eligibility was defined based on age and smoking-related criteria (pack-years and smoking cessation interval). Stage distribution is reported among eligible patients with known stage and reflects the proportion of early-stage (Stage I–II) versus advanced-stage (Stage III–IV) disease.

Scenario	Category	n	%
USPSTF	Eligible	431	24.38
	Not eligible	1337	75.62
	Stages I–II among eligible	43	18.78
	Stages III–IV among eligible	186	81.22
NCCN	Eligible	453	25.62
	Not eligible	1315	74.38
	Stages I–II among eligible	45	18.60
	Stages III–IV among eligible	197	81.40
ERS	Eligible	396	22.40
	Not eligible	1372	77.60
	Stages I–II among eligible	39	18.48
	Stages III–IV among eligible	172	81.52

## Data Availability

The data underlying this study are derived from institutional clinical records and are not publicly available due to privacy and regulatory constraints. Deidentified data may be made available upon reasonable request to the corresponding author, subject to institutional approval. All analytical procedures and statistical workflows can be provided upon request to ensure reproducibility.

## Supplementary Materials

The following supporting information can be downloaded at: https://www.mdpi.com/article/10.3390/diagnostics16172849/s1, Table S1: Sensitivity analysis of guideline-based screening eligibility under alternative assumptions for missing smoking and pack-year information.

## Abbreviations

The following abbreviations are used in this manuscript:

Abbreviation	Definition
CEE	Central and Eastern Europe
NLST	National lung screening trial
NELSON	Dutch–Belgian lung cancer screening trial
NSCLC	Non-small cell lung cancer
SCLC	Small cell lung cancer
SCC	Squamous cell carcinoma
EGFR	Epidermal growth factor receptor
KRAS	Kirsten rat sarcoma viral oncogene homolog
ALK	Anaplastic lymphoma kinase
PD-L1	Programmed death-ligand 1
COPD	Chronic obstructive pulmonary disease
TB	Tuberculosis
LDCT	Low-dose computed tomography
VDT	Volumetric/Volume doubling time
miRNA	MicroRNA
IOB	Institute of Oncology Bucharest
TNM	Tumor, node, metastasis
pTNM	Pathological tumor, node, metastasis
RT	Radiotherapy
CHT	Chemotherapy
SD	Standard deviation
IQR	Interquartile range
USPSTF	United States Preventive Services Task Force
NCCN	National Comprehensive Cancer Network
ERS	European Respiratory Society
MOI	Missed opportunity index
PLCOm2012	Prostate, lung, colorectal and ovarian cancer screening trial model 2012
PM	Particulate matter
PM10	Particulate matter ≤10 µm
PM2.5	Particulate matter ≤2.5 µm
PAHs	Polycyclic aromatic hydrocarbons
