# Supplementary material for: Late-Stage Presentation and Diagnostic Gaps in Romanian Lung Cancer Patients: A Retrospective Tertiary Center Analysis with Implications for Scalable Screening
Source: Diagnostics (Basel). 2026 Sep 4;16(17):2849. doi: 10.3390/diagnostics16172849 (PMC13565030; doi:10.3390/diagnostics16172849)
Supplement: Supplementary file 1 [file diagnostics-16-02849-s001.zip › diagnostics-4251000-supplementary.pdf]

Late-Stage Presentation and Diagnostic Gaps in Romanian Lung Cancer Patients: A Retrospective Tertiary Center Analysis with Implications for Scalable Screening

Liviu Bîlteanu [1,2,3], Antonia-Ruxandra Folea [2,*], Vlad-Luca Moga [2], Bogdan Cosmin Tănase [4], Steluța Bărăscu [4], Cristian Pavel [2], Diana Troncotă [2], Matei Celea [2], Octavian Buiu [3], Andreea-Iren Șerban [1,5] and Rodica Anghel [2]

1 Faculty of Biology, University of Bucharest, 91-95 Splaiul Independentei, 050095 Bucharest, Romania; liviu.bilteanu@umfcd.ro (L.B.); andreea-iren.serban@fmvb.usamv.ro (A.-I.Ș.)
2 Faculty of General Medicine, Carol Davila University of Medicine and Pharmacy, 8 Eroii Sanitari Street, 050474 Bucharest, Romania; vlad-luca.moga@drd.umfcd.ro (V.-L.M.); cristian-pavel.pavel@drd.umfcd.ro (C.P.); diana.troncota@drd.umfcd.ro (D.T.); mateicelea@gmail.com (M.C.); rodica.anghel@umfcd.ro (R.A.)
3 Laboratory for Molecular Nanotechnologies, National Institute for Research and Development in Microtechnologies—IMT Bucharest, 126A, Erou Iancu Nicolae Street, 077190 Voluntari, Romania; octavian.buiu@imt.ro (O.B.)
4 Oncological Institute "Alexandru Trestioreanu" Bucharest, 252 Soseaua Fundeni, 022328 Bucharest, Romania; bogdansen@gmail.com (B.C.T.); stelutahuzuu@yahoo.com (S.B.)
5 Department of Preclinical Sciences, Faculty of Veterinary Medicine, University of Agronomic Sciences and Veterinary Medicine, 105 Splaiul Independentei, 050097 Bucharest, Romania
* Correspondence: antonia-ruxandra.folea@drd.umfcd.ro

Supplementary Material

Supplementary Table S1. Sensitivity analysis of guideline-based screening eligibility under alternative assumptions for missing smoking and pack-year information.

Guideline	Missing-data scenario	Eligibility denominator N	Eligible n	Eligible %	Not eligible n	Not eligible %	Early-stage capture %	MOI %
USPSTF	Primary conservative	1,768	431	24.38	1,337	75.62	30.07	69.93
USPSTF	Available-case	684	431	63.01	253	36.99	69.35	30.65
USPSTF	Worst-case bound	1,768	431	24.38	1,337	75.62	30.07	69.93
USPSTF	Best-case bound	1,768	1,515	85.69	253	14.31	86.71	13.29
NCCN	Primary conservative	1,768	453	25.62	1,315	74.38	31.47	68.53
NCCN	Available-case	584	453	77.57	131	22.43	86.54	13.46
NCCN	Worst-case bound	1,768	453	25.62	1,315	74.38	31.47	68.53
NCCN	Best-case bound	1,768	1,637	92.59	131	7.41	95.10	4.90
ERS	Primary conservative	1,768	396	22.40	1,372	77.60	27.27	72.73

Guideline	Missing-data scenario	Eligibility denominator N	Eligible n	Eligible %	Not eligible n	Not eligible %	Early-stage capture %	MOI %
ERS	Available-case	779	396	50.83	383	49.17	56.52	43.48
ERS	Worst-case bound	1,768	396	22.40	1,372	77.60	27.27	72.73
ERS	Best-case bound	1,768	1,385	78.34	383	21.66	79.02	20.98

The primary conservative analysis classifies indeterminate eligibility due to missing essential smoking-related information as not eligible. The available-case analysis excludes indeterminate patients from the denominator. The worst-case bound assigns all indeterminate cases as not eligible. The best-case bound assigns indeterminate cases as eligible only when observed non-missing guideline components do not already establish ineligibility. USPSTF is implemented approximately because exact years since smoking cessation are unavailable; documented former smokers are therefore accepted for the smoking-status component. NCCN uses the available >=20 pack-year branch because validated total smoking duration is unavailable. MOI = early-stage patients not eligible divided by all early-stage patients included in the scenario-specific eligibility denominator.
